# Persistent Zn^2+^ Influx‐Mediated Deacetylation of ANXA2 Regulates the “Zn^2+^‐Autophagic Flux” and Alleviates Intervertebral Disc Degeneration

**DOI:** 10.1002/advs.77870

**Published:** 2026-09-27

**Authors:** Yuxin Jin, Yongcheng Chen, Yihao Xie, Dongyang Hu, Longhui Gong, Zhihua Chen, Linjie Chen, Minhao Gao, Qizhu Chen, Morgan Jones, Hui Wang, Xiuling You, Kenny Yat Hong Kwan, Shoutao Weng, Taidong Lyu, Xiang Chen, Yuxiao Zhu, Yusheng Wang, Bin Li, Xiangyang Wang, Xiaofeng Jia, Kailiang Zhou, Ouqiang Wu, Aimin Wu

**Affiliations:** ^1^ Department of Orthopaedics Zhejiang‐Hong Kong Joint Laboratory for Precision Diagnosis and Treatment of Spinal Diseases Key Laboratory of Orthopaedics of Zhejiang Province The Second Affiliated Hospital and Yuying Children's Hospital of Wenzhou Medical University Wenzhou Zhejiang P. R. China; ^2^ Department of Clinic of Spine Center Xinhua Hospital Shanghai Jiaotong University School of Medicine Shanghai P. R. China; ^3^ Spine Unit The Royal Orthopaedic Hospital Northfield Birmingham UK; ^4^ Department of Orthopaedics and Traumatology Li Ka Shing Faculty of Medicine The University of Hong Kong Pokfulam Hong Kong; ^5^ Orthopedic Institute Department of Orthopaedic Surgery The First Affiliated Hospital School of Biology & Basic Medical Sciences Suzhou Medical College Soochow University Suzhou Jiangsu P. R. China; ^6^ Department of Neurosurgery Orthopaedics Neurobiology University of Maryland School of Medicine Baltimore Maryland USA; ^7^ Department of Orthopaedics Key Laboratory of Musculoskeletal System Degeneration and Regeneration Translational Research of Zhejiang Province Sir Run Shaw Hospital Zhejiang University School of Medicine Hangzhou P. R. China

**Keywords:** acetylation, autophagy, IVDD, mitochondrion, Zn2+

## Abstract

Intervertebral disc degeneration (IVDD) is an age‐related disease accompanied by disrupted mitochondrial homeostasis and defective autophagy. Cellular senescence induces ion imbalance, which leads to mitochondrial damage and suppressed autophagic flux; nevertheless, the molecular mechanism underlying this process remains poorly understood. Here, by integrating single‐cell RNA sequencing (single‐cell RNA‐seq), messenger RNA sequencing (mRNA‐seq), and liquid chromatography‐mass spectrometry (LC‐MS), we identified zinc transporter ZIP14 (SLC39A14) as a critical regulatory factor. Further investigations revealed that deacetylation at lysine 302 (K302) of annexin A2 (ANXA2) serves as a core intracellular zinc‐dependent regulatory node controlling mitophagy. In vitro site‐directed mutagenesis combined with molecular dynamics simulations demonstrated that acetylation at the ANXA2 K302 residue alters the conformation and function of the ANXA2‐mTOR complex. Specifically, K302 acetylation enhances the interaction between ANXA2 and mTOR, consequently restraining cellular mitophagy. Mechanistically, zinc‐dependent modification of ANXA2 governs mTOR activity, bridging disturbed ion homeostasis and autophagy machinery. We also characterized the intracellular degradation pattern of ANXA2 and clarified its abnormal accumulation during IVDD development. In vivo experiments verified that ZIP14 (SLC39A14) is required to maintain intracellular Zn^2^^+^ levels and sustain ANXA2‐mTOR signaling. Consistently, dietary zinc supplementation effectively slows IVDD progression, highlighting a potential therapeutic strategy.

## Graphical abstract

Zinc transporter SLC39A14 sustains intracellular Zn^2^^+^ levels to activate SIRT3‑dependent ANXA2 deacetylation at K302. This modification weakens ANXA2‑mTOR binding and releases autophagic‑flux suppression in nucleus pulposus cells. Dysregulated Zn^2^^+^ homeostasis disrupts this axis to drive intervertebral disc degeneration.

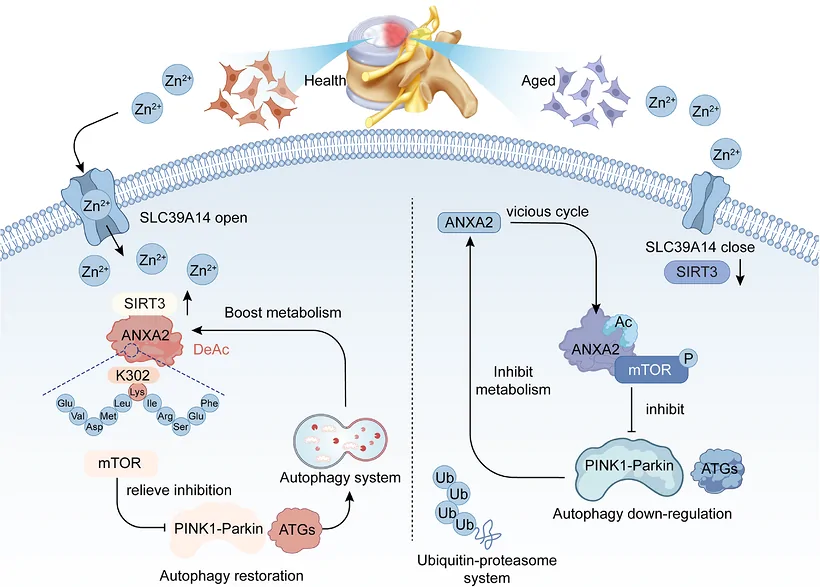

## 1 Introduction

Intervertebral disc degeneration (IVDD) represents the primary pathological driver of chronic low back pain, which affects approximately 80% of adults at some stage during their lifetime and ranks among the leading causes of years lived with disability worldwide [1, 2]. Patients with IVDD commonly suffer from persistent axial back pain, radiating limb numbness and pain, and limited spinal mobility [3]. Radiologically, IVDD manifests as intervertebral space narrowing, endplate sclerosis, disc herniation, and Modic changes [4]. Due to its high lifetime prevalence and the absence of disease‐modifying therapeutic strategies, IVDD results in enormous medical expenditures and work absenteeism, creating a heavy global socioeconomic burden [5]. The core pathological hallmarks of IVDD encompass progressive extracellular matrix breakdown, nucleus pulposus cell senescence, and aberrant inflammatory cascades, which are widely triggered by oxidative stress and mitochondrial dysfunction [6, 7]. At present, available therapeutic options range from conservative treatments (analgesics, physical therapy) to invasive surgery; nevertheless, these strategies only achieve symptomatic relief and cannot arrest the underlying disc degenerative process [8, 9]. Therefore, elucidating novel molecular pathways that regulate intervertebral disc homeostasis and degeneration remains an urgent need.

As the core structure for spinal movement and load transmission, the intervertebral disc represents the largest avascular tissue in the human body and possesses unique immune‐privileged properties. It is composed of three discrete compartments: the nucleus pulposus (NP), annulus fibrosus (AF), and cartilaginous endplates (CEP). It resides in a specialized microenvironment that is relatively avascular, hypoxic, and rich in proteoglycans [10, 11, 12]. In this context, ion transport—particularly metal ions such as zinc (Zn^2^^+^) and calcium (Ca^2^^+^)—helps maintain osmotic and cellular homeostasis and can also function as signaling cues [13, 14]. Changes in ion homeostasis can increase the response threshold of intervertebral disc cells to osmotic stress and inflammatory stimuli, thereby affecting the functional state of mitochondria and other organelles, including mitochondrial damage [15]. Even slight deviations in metal ion homeostasis can thus be amplified into cascading changes in signal transduction and metabolic adaptation, laying the groundwork for subsequent mitochondrial dysfunction.

During the progression of IVDD, the protective dynamic balance of autophagy is disrupted [16]. Metal ion signals are directly integrated into autophagy regulation: Zn^2^^+^ levels affect the autophagic initiation threshold of the Akt/mTOR axis [17] and participate in autophagosome‐lysosome fusion as well as lysosomal acidification and enzyme activity mediated by SNARE proteins (e.g., STX17‐VAMP8). Dysregulation of metal ion flux therefore not only increases cellular damage but also constrains autophagic flux, simultaneously limiting both damage generation and clearance, creating a positive feedback loop for degeneration [18]. Under this stress‐induced autophagic dysregulation, mTORC1 and AMPK sense cellular nutritional status and energy levels, respectively, and regulate autophagy through ULK1 and class III PI3K complex I [19].

This study combined single‐cell RNA sequencing (single‐cell RNA‐seq), messenger RNA sequencing (mRNA‐seq), and liquid chromatography‐mass spectrometry (LC‐MS). We focus on Zn^2^^+^‐dependent modulation of mitophagy and autophagy, as well as homeostasis disturbance in nucleus pulposus cells (NPCs). We explored whether the Zn^2^^+^ influx transporter SLC39A14 (ZIP14) serves as a key regulator in IVDD. As a member of the SLC39 family, SLC39A14 mediates extracellular Zn^2^^+^ uptake and intracellular Zn^2^^+^ redistribution toward mitochondria [20]. Zn^2^^+^ can further support mitochondria‐associated protective signaling through Sirt3‐related mechanisms [21]. Accordingly, we aimed to clarify how Zn^2^^+^ depletion induced by SLC39A14 downregulation modulates the ANXA2‐mTOR complex, and whether it causes concurrent damage to mitochondrial function and autophagy, ultimately leading to NPC homeostasis imbalance and IVDD progression.

This study explored the mechanisms underlying NPC homeostasis imbalance in IVDD, with four core research objectives: (1) whether the cascade of “aging → SLC39A14 downregulation → Zn^2^^+^ depletion → dual impairment of mitochondrial function and autophagy” is the key pathological mechanism responsible for NPC homeostasis imbalance in age‐related IVDD; (2) exploration of the core mechanism by which Zn^2^^+^ regulates the structure and function of the ANXA2‐mTOR complex; (3) whether the ANXA2 metabolic negative feedback triggered by autophagy inhibition via the ANXA2‐mTOR complex plays a crucial role in the fast progression of advanced IVDD; (4) investigating the impact and safety of zinc supplementation in diets for delaying IVDD in animal models.

## 2 Results

### 2.1 SLC39A14 Deficiency and Sustained Zn^2^^+^ Depletion in Senescent NPCs

**FIGURE 1:**
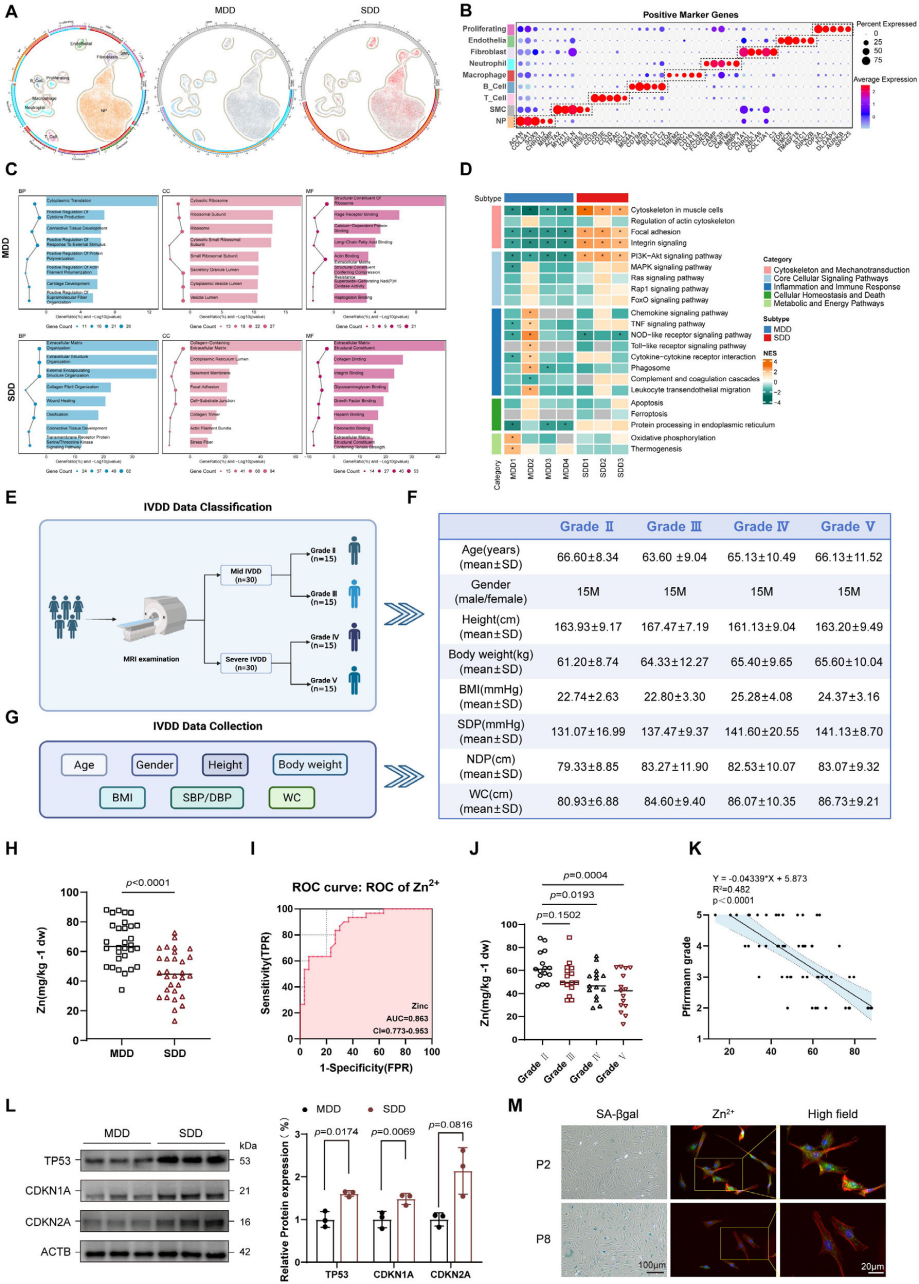
Detection of SLC39A14 protein expression and Zn^2^^+^ content in degenerated nucleus pulposus (NP). (A) UMAP visualized the cellular clustering of the human tissue samples. (B) The bubble plot shows the marker genes of each cell subcluster. (C) Comparative GO analysis between MDD and SDD. (D) Comparative KEGG analysis between MDD and SDD. (E) Data Classification and Labeling for Patients with IVDD. (F) Patient characteristics of each IVDD Grade. (G) Collect information on patients' age, gender, height, body weight, Body Mass Index (BMI), Systolic Blood Pressure / Diastolic Blood Pressure (SBP/DBP), and waist circumference (WC). (H) Quantified Zn^2^^+^ content in human NP tissue between the mid‐IVDD group and the IVDD degeneration group (n = 30 per group). (I) Zn^2^^+^ level in human NP tissue for distinguishing IVDD patients from healthy individuals: ROC curve (AUC = 0.863) (n = 30 per group). (J) Quantified Zn^2^^+^ content in human NP tissue samples graded II–V according to the Pfirrmann classification (n = 15 per group). (K) Spearman correlation analysis between human NP tissue Zn^2^^+^ content and Pfirrmann grade (R^2^ = 0.482, *p* < 0.0001) (n = 15 per group). (L) WB analysis showed the protein levels of TP53, CDKN1A, and CDKN2A in intervertebral discs from the control and degenerated groups (n = 3). (M) Senescence‐associated β‐galactosidase (SA‐β‐gal) staining (n = 6, Scale bar: 100 µm) and intracellular Zn^2^^+^ immunofluorescence staining (n = 6, Scale bar: 20 µm) of human NPCs at passage 2 (P2) and passage 8 (P8).

Aging is a complex physiological process associated with disordered trace element homeostasis. Zinc is indispensable for the human body, yet it remains unclear whether disrupted zinc homeostasis drives progressive degeneration of intervertebral disc NPCs. Herein, human nucleus pulposus tissues were divided into four groups (Pfirrmann grades II‐V) according to preoperative lumbar MRI and Pfirrmann grading standards (Table S1). Meanwhile, the single‐cell transcriptome dataset GSE244889 downloaded from the public GEO database was uniformly analyzed and categorized into moderate (MDD, Pfirrmann grades II‐III) and severe (SDD, Pfirrmann grades IV‐V) intervertebral disc degeneration groups. Nine cell subpopulations including NPCs were identified, with NPCs being the dominant cell type (Figure 1A,B). MDD samples were mainly enriched in peripheral immune cells, while SDD samples were dominated by intrinsic NPCs. According to Gene Ontology (GO) enrichment analysis, the DEGs of MDD were predominantly engaged in cytoplasmic translation, ribosome structure, and cytokine regulation, implying early‐to‐moderate disc degeneration correlates with inflammation and metabolic disturbance. By contrast, SDD‐related differentially expressed genes (DEGs) were enriched in extracellular matrix organization, collagen synthesis, wound healing, and cell adhesion, revealing close correlations with matrix remodeling, abnormal collagen metabolism, and matrix homeostasis imbalance (Figure 1C). Combined Kyoto Encyclopedia of Genes and Genomes (KEGG) and Gene Set Enrichment Analysis (GSEA) analyses indicated that NPCs at severe degeneration stage activated matrix remodeling and fibrotic pathways such as focal adhesion, integrin signaling, and PI3K‐Akt pathway (Figure 1D).

To further investigate whether the degree of disease degeneration is associated with trace element levels, demographic and clinical variables of the patients were collected, including age, gender, body weight, body mass index (BMI), systolic/diastolic blood pressure (SBP/DBP), and waist circumference (WC). The baseline characteristics of the patients were presented in tabular form and showed no significant differences between groups (Figure 1E–G). We then measured trace element levels (Zn, Fe, Cu, Mg, Ca) in the serum of patients from the control and degenerative groups and compared the Mid‐IVDD versus Severe‐IVDD groups. Among these elements, serum Zn showed the strongest association with IVDD: ROC analysis yielded an area under the curve (AUC) of 0.807, and Spearman correlation analysis gave R^2^ = 0.377 (Figure S1A,B). Serum Fe: ROC‐AUC = 0.759, R^2^ = 0.268 (Figure S1C,D); serum Cu: ROC‐AUC = 0.592, R^2^ = 0.030 (Figure S1E,F); serum Mg: ROC‐AUC = 0.666, R^2^ = 0.131 (Figure S1G,H); serum Ca: ROC‐AUC = 0.620, R^2^ = 0.010 (Figure S1I,J). These findings show a moderate relationship between serum zinc levels and IVDD. Notably, this correlation does not confirm a direct causal or mechanistic relationship, and further experimental validation is required to clarify underlying mechanisms. The Zn^2^^+^ concentration in NP tissues was found to be significantly lower in the IVDD group than in the control group, according to atomic absorption spectrometry. ROC curve analysis showed that NP tissue Zn^2^^+^ content had discriminative value for IVDD occurrence (AUC = 0.863, *p* < 0.0001) (Figure 1H,I). Zn^2^^+^ content decreased progressively with higher Pfirrmann grades (Figure 1J), and Spearman correlation analysis indicated that as Zn^2^^+^ content increases, the degeneration grade decreases (R^2^ = 0.482, *p* < 0.0001) (Figure 1K). Western blot analysis showed that senescence markers (TP53, CDKN1A, and CDKN2A) were significantly upregulated in degenerated NP tissues compared to the low‐grade group (Figure 1L). To further examine the relationship between cellular senescence and intracellular Zn^2^^+^ content, we conducted senescence‐associated β‐galactosidase (SA‐β‐gal) and Zn^2^^+^ immunofluorescence staining on human NPCs at passage 2 (P2) and passage 8 (P8). Spatial colocalization analysis demonstrated that intracellular Zn^2^^+^ levels decreased as the proportion of senescent cells increased (Figure 1M).

Zn^2^^+^ homeostasis depends on coordinated regulation by multiple transports, particularly the ZIP (SLC39A family) and ZnT (SLC30A family) transporter families [22, 23]. We compared mRNA transcription levels of Zn^2^^+^ transport‐related genes in NP tissues from three healthy individuals (control) and three patients with disc degeneration (IVDD group) (GSE186542). Heatmap and volcano plot analyses led to the identification of the SLC39A and SLC30A families. Finally, we screened out SLC39A14 as the target gene with the highest expression level in the control group and the most significant downregulation trend in the degenerative group (Figures S2A and S2B). Gene Ontology (GO) analysis between the IVDD group and the control group revealed that the enriched terms were mainly involved in Zinc & Ion Homeostasis and Mitochondrial Stress & Hypoxia (Figure S2C). Subsequently, by comparing the mRNA and protein expression levels as well as the tissue fluorescence intensity of SLC39A14 between the control group and the degenerative group, the downregulation trend of this gene was further confirmed. (Figure S2D–F). To model senescence and mitochondrial damage in vitro, NPCs were treated with tert‐butyl hydroperoxide (TBHP), resulting in decreased SLC39A14 transcription and protein expression (Figure S2G,H), accompanied by reduced intracellular Zn^2^^+^ content (Figure S2K). Following identification of SLC39A14 as a key Zn^2^^+^ transporter, we constructed a lentivirus‐loaded short hairpin RNA (shRNA) targeting SLC39A14 (sh‐SLC39A14) (Table S5), which achieved efficient silencing (Figure S2I,J). Cells transfected with sh‐SLC39A14 exhibited lower intracellular Zn^2^^+^ levels than controls (Figure S2L).

Overall, our results confirm that both Zn^2^^+^ transporter expression and intracellular Zn^2^^+^ levels are significantly downregulated in senescent or degenerated NP tissues and their resident cells. This downregulation is strongly associated with cellular senescence and provides a theoretical foundation for further studies on Zn^2^^+^ influx and its role in preventing and treating IVDD.

### 2.2 SLC39A14‐Mediated Zn^2^^+^ Flux is Essential to Alleviate Mitochondrial Dysfunction and Delay Cellular Senescence

**FIGURE 2:**
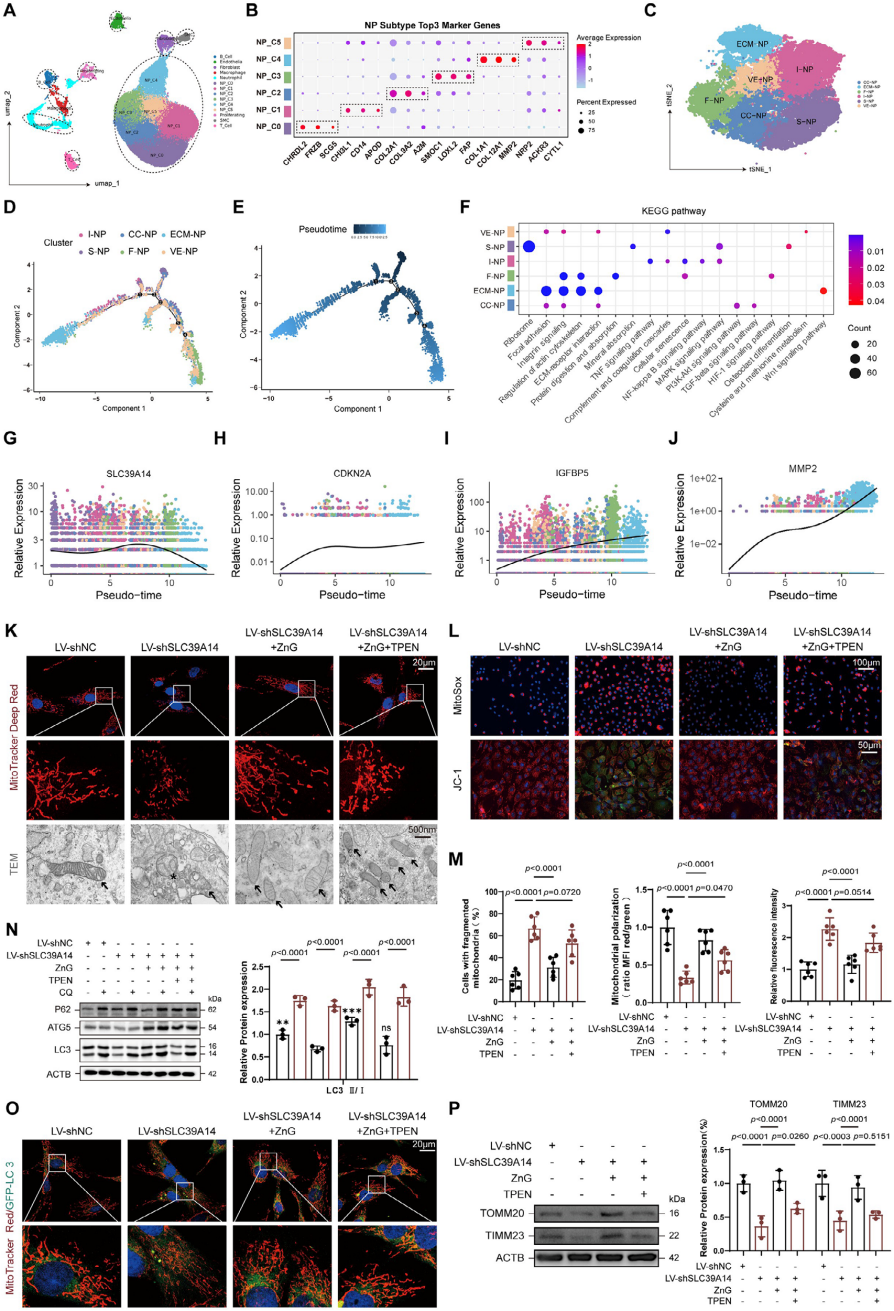
Inactivation of SLC39A14 induces senescence and mitochondrial damage in NPCs. (A) UMAP visualization showing six refined subclusters within human NPC. (B) NP Subtype Top3 Marker Genes. (C) t‐SNE plot illustrating the distribution of S‐NP, I‐NP, VE‐NP, CC‐NP, F‐NP, and ECM‐NP subtypes. (D) Pseudotime trajectory colored by distinct cell subtypes. (E) Pseudotime trajectory plot showing cells colored according to pseudotime progression. (F) KEGG pathway enrichment analysis of six NP cell subpopulations. (G–J) Dynamic expression patterns of SLC39A14, CDKN2A, IGFBP5, and MMP2 along Monocle‐derived pseudotime trajectories. Each dot denotes an individual cell, and cells are colored by cell subtypes. (K) MitoTracker staining and TEM showed mitochondrial health status in human NPCs with SLC39A14 silencing following different treatments. (n = 6; MitoTracker Scale bar: 20 µm; TEM Scale bar: 500 nm, arrows indicate mitochondria, and asterisks mark autophagosomes). (L) MitoSOX and JC‐1 staining of intracellular ROS levels and mitochondrial membrane potential (Δψm) after different treatments (n = 6; MitoSOX Scale bar: 100 µm; JC‐1 Scale bar: 50 µm). (M) Quantitative analysis of mitochondrial fragmentation, mitochondrial membrane potential, and relative MitoSOX fluorescence intensity (n = 6). (N) Quantitative analysis of LC3‐II/LC3‐I, p62, and ATG5 under different treatment conditions (** = 0.0286, *** = 0.0007, ns = 0.7385; n = 3). (O) Double fluorescence staining with MitoTracker and GFP‐LC3 to evaluate mitophagy levels (n = 6; Scale bar: 20 µm). (P) Comparison of mitochondrial membrane proteins (TOMM20, Translocase of Inner Mitochondrial Membrane 23) among different treatment groups (n = 3). Note: mitophagy flux (LC3 II/I+ CQ, GFP‐LC3 + CQ); static marker levels (Mitotracker Red, TEM, MitoSOX, JC‐1, TOMM20 and TIMM23).

To dissect NPC heterogeneity, we identified six distinct sub‐clusters (NP_C0‐C5) and functionally annotated them based on their top three specific marker genes (Figure 2A; Figure S3A). These included Secretory‐NPCs (S‐NP; CHRDL2/FRZB/SCG5 [24, 25, 26]), Inflammatory‐NPCs (I‐NP; CHI3L1/CD14/APOD [27, 28, 29]), Chondrogenic‐NPCs (CC‐NP; COL2A1/COL9A2/A2M [30, 31, 32]), Fibrous‐type NPCs (F‐NP; SMOC1/LOXL2/FAP [33, 34, 35]), ECM‐remodeling NPCs (ECM‐NP; COL1A1/COL12A1/MMP2 [36, 37, 38]), and Vascular NPCs (VE‐NP; NRP2/ACKR3/CYTL1 [39, 40, 41]) (Figure 2B,C). Pseudotime trajectory (Monocle 2) and KEGG enrichment analyses of these sub‐clusters revealed S‐NP as the ancestral root state. The trajectory demonstrated that, driven by concurrent inflammation (I‐NP) and vascularization (VE‐NP), the physiological chondrogenic phenotype (CC‐NP) gradually transitioned toward terminal fibrosis (F‐NP) and stiff extracellular matrix accumulation (ECM‐NP) (Figure 2D–F). Notably, gene expression profiling along the pseudotime trajectory revealed a continuous downregulation of the Zn^2+^ transporter SLC39A14 (Figure 2G). This decline strongly synchronized with the elevation of senescence (CDKN2A, IGFBP5) (Figure 2H,I) and catabolic (MMP2) (Figure 2J) markers, highlighting a critical link between SLC39A14 depletion and the senescent‐fibrotic fate of NPCs.

Zn^2^^+^ acts as a pivotal ion mediating cellular antioxidant stress, and mitochondria are core organelles highly susceptible to intracellular oxidative stress. We created a lentiviral vector containing a short hairpin RNA (shRNA) sequence targeting SLC39A14 (LV‐shSLC39A14) and introduced it into NPCs to further study the regulatory role of the SLC39A14 transporter in autophagy and mitochondrial morphology. To primarily assess mitochondrial structural integrity and static functional markers, we employed transmission electron microscopy (TEM) and specific mitochondrial probes. Observations using the mitochondrial probe MitoTracker combined with TEM showed that, compared with the empty vector group (LV‐shNC), SLC39A14 knockdown caused mitochondrial swelling, loss of cristae structure, and internal vacuolization. Fluorescence imaging revealed that mitochondria transformed from an elongated morphology to a short, curled shape (Figure 2K). Supplementation with zinc gluconate (ZnG, an exogenous Zn^2^^+^ source) under SLC39A14‐inhibited conditions partially reversed this mitochondrial damage, whereas the Zn^2^^+^ chelator TPEN antagonized the protective effect of ZnG.

Zinc ions block the opening of the mitochondrial permeability transition pore (mPTP), sustain the mitochondrial membrane potential (Δψm), and avert the release of cytochrome c, thereby suppressing excessive mitochondrial ROS production [42, 43]. Measurement of mitochondrial ROS and Δψm using MitoSOX and JC‐1 probes confirmed that SLC39A14 inhibition increased ROS levels and caused Δψm depolarization, indicating that intracellular Zn^2^^+^ is critical for restoring mitochondrial function (Figure 2L,M). These indices, serving as static markers, provided a baseline assessment of mitochondrial health.

Then, the expression levels of static autophagy‐related markers (LC3, ATG5, and P62) were assessed via western blot analysis. ZnG treatment increased LC3‐II and ATG5 expression and decreased P62 levels, indicating autophagy activation. These effects were partially blocked by co‐treatment with TPEN, and the ZnG‐induced increase in LC3‐II was further enhanced by co‐administration of chloroquine (CQ, a classic lysosomal inhibitor), confirming elevated autophagic flux (Figure 2N; Figure S3D). To monitor autophagic flux, we utilized the mCherry‐LC3/GFP‐LC3 dual‐labeled lentivirus system (Figure S3C,J), while FluoZin‐3 and RhodZin‑3 AM were used to measure the Zn^2^^+^ concentrations in the whole cytoplasm and mitochondria, respectively (Figure S3B,I). Silencing SLC39A14 reduced Zn^2^^+^ influx and inhibited autophagic flux. Double fluorescence labeling of MitoTracker and LC3, along with detection of TOMM20 and TIMM23, and western blot analysis, showed that SLC39A14 knockdown impaired mitochondrial membrane integrity and decreased autophagic flux. ZnG supplementation effectively mitigated this damage, while TPEN antagonized Zn^2^^+^‐mediated autophagy activation (Figure 2O,P; Figure S3E).

The senescence‐associated secretory phenotype (SASP) is a hallmark of cellular senescence. To investigate the role of SLC39A14‐mediated Zn^2^^+^ influx in SASP, senescence‐associated β‐galactosidase (SA‐β‐gal) staining was performed. Inhibition of SLC39A14 increased the proportion of senescence‐positive cells, while ZnG supplementation partially reversed this effect (Figure S3F). Zn^2^^+^ deficiency is closely associated with cellular senescence [44], but whether this depends on SLC39A14 remained unclear. Western blot analysis of senescence‐related proteins (TP53, CDKN1A, CDKN2A) and SASP factors (TNF‐α, IL‐1β, IL‐6) demonstrated that ZnG supplementation alleviated SASP upregulation induced by SLC39A14 knockdown (Figure S3G,H).

These findings indicate that SLC39A14‐mediated Zn^2^^+^ influx maintains autophagy levels in NPCs, concurrently delaying cellular senescence and inhibiting SASP secretion.

### 2.3 Role of SLC39A14 and Zn^2^^+^ Content in Maintaining Intervertebral Disc Integrity in Aged Sprague‐Dawley Rats

**FIGURE 3:**
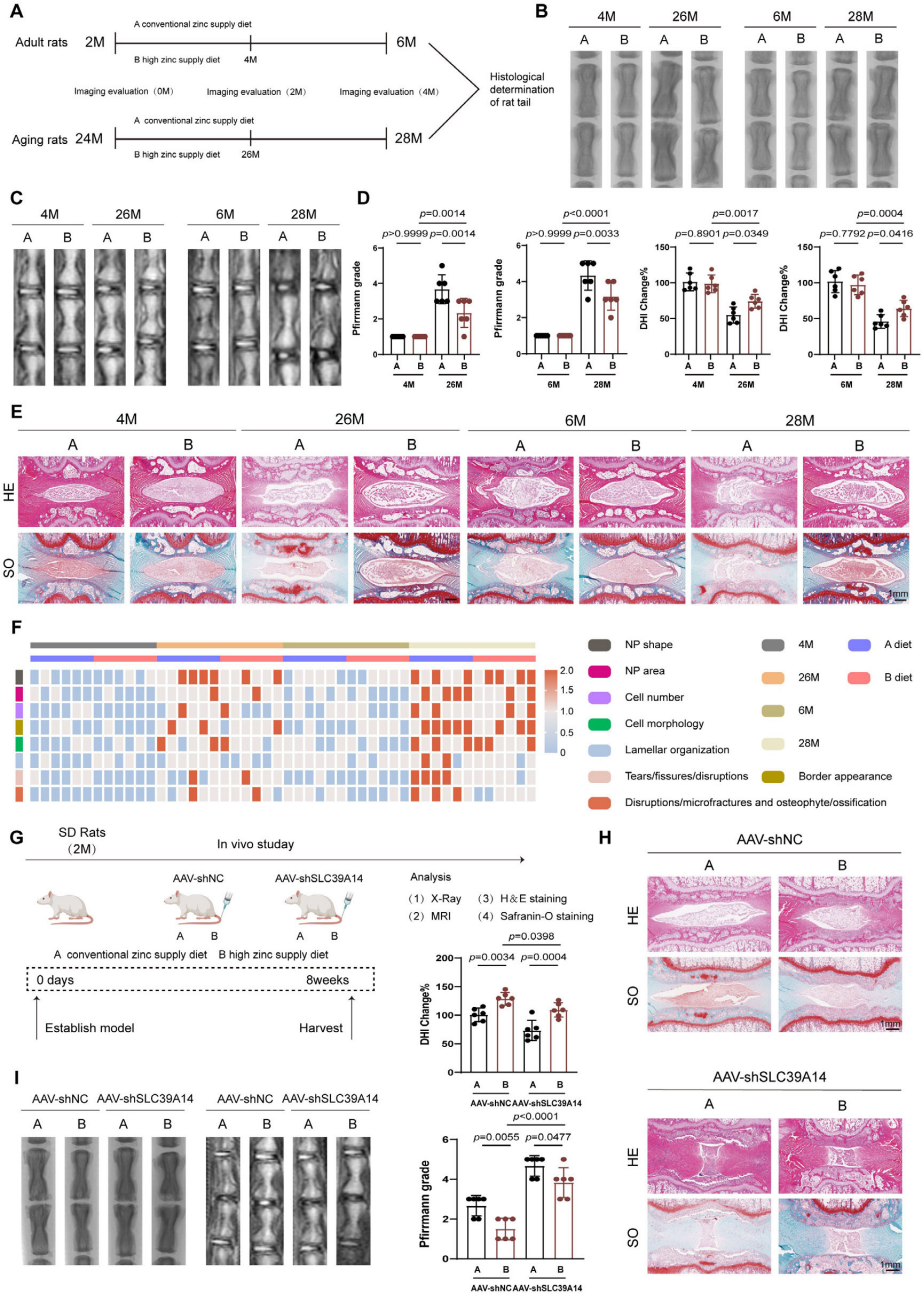
Efficacy of SLC39A14 and Zn^2^^+^ in maintaining intervertebral disc morphology in rats. (A) Schematic diagram of the feeding strategy for aged rats receiving a regular‐zinc diet and a high‐zinc diet. (B) Comparison of caudal intervertebral disc height in aged rats after 4 months of different dietary treatments (n = 6). (C) Comparison of MRI images of caudal intervertebral discs in aged rats after 4 months of different dietary treatments (n = 6). (D) Quantification of disc height and NP integrity using the DHI score and Pfirrmann score (n = 6). (E) HE and SO staining of caudal intervertebral disc sections from rats after 4 months of different feeding strategies (n = 6; Scale bar: 1 mm). (F) Quantitative analysis of data (based on NP shape, NP area, cell number, cell morphology, border appearance, lamellar organization, tears/fissures/disruptions, disruptions/microfractures, and osteophyte/ossification) was visualized as a heatmap (n = 6). (G) Schematic diagram of regular‐zinc and high‐zinc feeding strategies after SLC39A14 silencing in a rat model of needle puncture‐induced NP injury. (H) HE and SO staining of caudal vertebral sections from rats after 8 weeks of different treatment strategies (n = 6; Scale bar: 1 mm). (I) Evaluation of intervertebral disc height and NP integrity using X‐ray and MRI after 8 weeks of different treatment strategies (n = 6).

Zn^2^^+^ protects against NPC senescence and mitochondrial damage. To further explore its functions in vivo and the role of SLC39A14, we used 2‐month‐old and 24‐month‐old Sprague‐Dawley (SD) rats. After adaptive feeding, rats were divided into two dietary groups: Group A received a regular zinc‐containing diet (zinc gluconate at 25 mg/kg), and Group B received a high‐zinc diet (zinc gluconate at 120 mg/kg) (Figure 3A). This dose did not cause damage to the internal organs of rats (Figure S4A).

During the intervention, X‐rays, MRI examinations, and histological sampling were performed at 2 and 4 months. X‐rays evaluated tail discs, and the disc height index (DHI) was measured (Figure S4G). The age‐related decrease in intervertebral disc height was significantly slower in the high‐zinc group than in the regular‐zinc group (Figure 3B,D). MRI T2‐weighted imaging showed that NP water content declined more slowly in high‐zinc 28‐month‐old rats compared with age‐matched controls (Figure 3C,D). HE and safranin O (SO) staining confirmed that zinc supplementation improved morphological integrity, cell density, and histological scores of NPCs (Figure 3E). Comprehensive NP, AF, and CEP scores were visualized as a heatmap (Figure 3F).

To examine the in vivo role of SLC39A14, adeno‐associated virus (AAV)‐mediated shRNA (sh‐SLC39A14) was used for local knockdown in caudal intervertebral discs. Rats were further divided into high‐zinc and regular‐zinc groups to investigate the effect of SLC39A14 silencing on exogenous zinc uptake and disc zinc homeostasis (Figure 3G). X‐ray and MRI showed that SLC39A14 knockdown partially attenuated the protective effect of zinc supplementation, evidenced by increased disc height loss and NP structural damage (Figure 3H,I). HE and SO staining confirmed that exogenous Zn^2^^+^ repair of NP tissue was limited in SLC39A14‐knockdown animals; the high‐zinc diet provided only slight structural improvement compared with the regular‐zinc diet (Figure 3H). Overall, these in vivo experiments demonstrate that exogenous Zn^2^^+^ supplementation delays IVDD in SD rats, but this protective effect partially depends on SLC39A14‐mediated Zn^2^^+^ transport.

### 2.4 SIRT3 Acts as a Binding Target for Sustained Intracellular Zn^2^^+^ Influx and Regulates the Function of Downstream ANXA2 Protein

**FIGURE 4:**
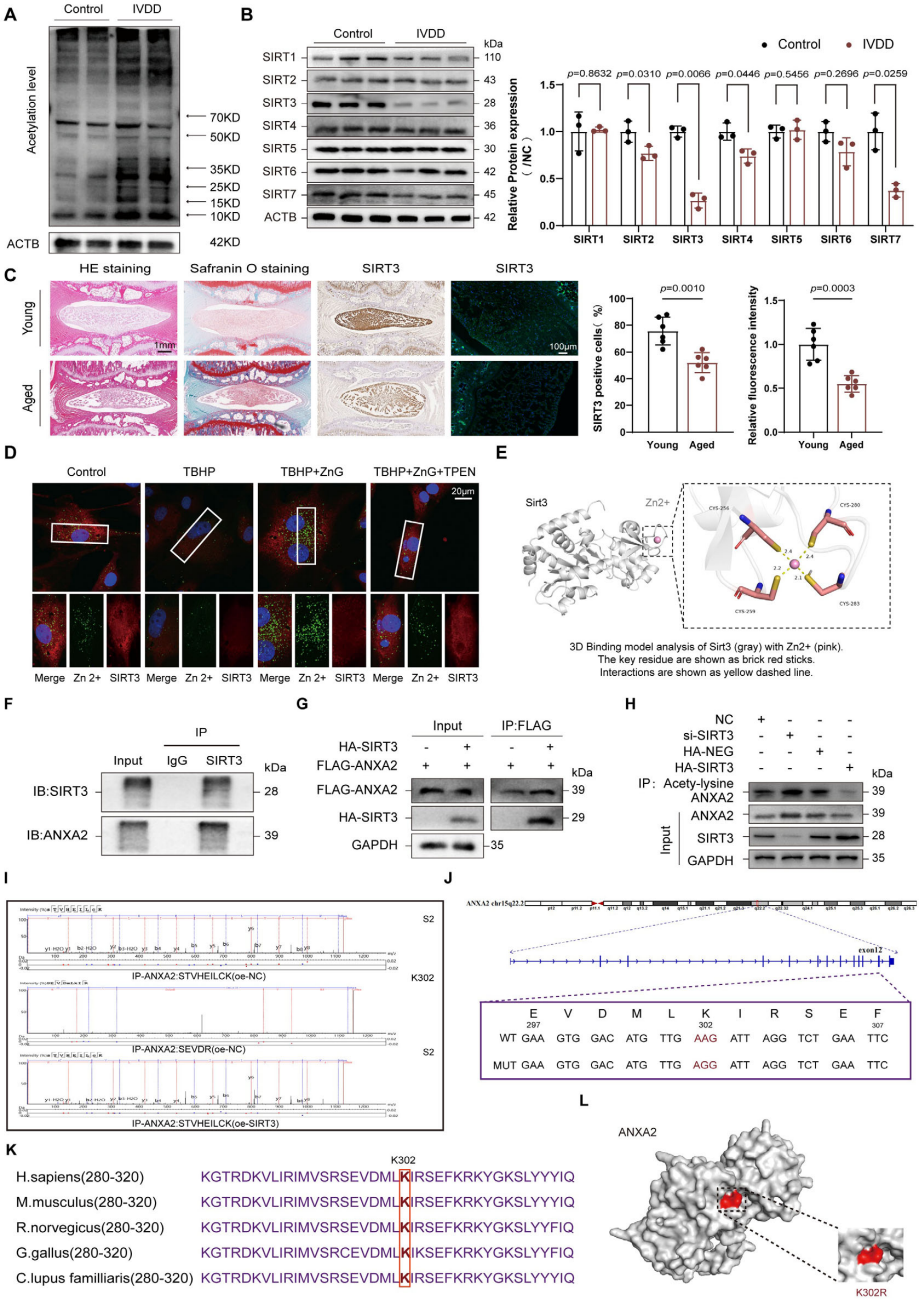
Efficacy of SIRT3 as a deacetylase in maintaining mitochondrial function. (A) Total acetylation levels detected in human NPCs from the control and degenerative groups (n = 3). (B) Protein levels of the sirtuin (SIRT) family in the control and degenerated group (n = 3). (C) HE staining, SO staining, and tissue expression level of SIRT3 in caudal sections from young and aged rats (n = 6; scale bar: 1 mm for HE, SO staining, and immunohistochemistry; 100 µm for tissue immunofluorescence). (D) Immunofluorescence staining of intracellular SIRT3 and Zn^2^^+^ after tert‐butyl hydroperoxide (TBHP) treatment and ZnG supplementation (n = 6; Scale bar: 20 µm). (E) Visualization and analysis of molecular docking sites between SIRT3 and Zn^2^^+^. (F) Co‐IP validated the endogenous interaction between SIRT3 and ANXA2 (n = 3). (G) Co‐IP confirming the exogenous binding between SIRT3 and ANXA2 (n = 3). (H) Detection of ANXA2 acetylation level (Ac‐ANXA2) after SIRT3 silencing or exogenous addition of HA‐SIRT3 (n = 3). (I) Changes in ANXA2 acetylation sites before and after SIRT3 overexpression (n = 3). (J) The codon of the K302 site was mutated to arginine via substitution of “A” with “G.” (K) The K302 site of ANXA2 is conserved across multiple species. (L) Molecular simulation surface showing the mutation site (K302R).

In the experiments described earlier, we demonstrated that the opening of the membrane ion channel SLC39A14 is a critical prerequisite for promoting sustained Zn^2^^+^ influx. We further revealed the importance of Zn^2^^+^ influx in regulating cellular physiological activities and alleviating oxidative stress. First, total protein acetylation levels were measured in NPCs isolated from human nucleus pulposus tissue from the control and IVDD groups (Figure 4A). IVDD cells exhibited significantly increased total protein acetylation. Among classic sirtuin (SIRT) family deacetylases, SIRT2, SIRT3, SIRT4, and SIRT7 were partially downregulated, with the reduction in SIRT3 being particularly notable (Figure 4B). In animal experiments, HE and SO staining of caudal intervertebral disc sections from young and aged rats, combined with immunohistochemical and immunofluorescence staining for SIRT3, revealed that aged rats exhibited increased gaps and decreased NP tissue density, accompanied by reduced SIRT3 staining and fluorescence intensity (Figure 4C). ZnG supplementation in cells from control and IVDD groups reduced total protein acetylation and increased SIRT3 expression among the SIRT family (Figure S4C,D). Similarly, high‐zinc feeding in rats upregulated SIRT3 expression in caudal disc sections (Figure S4B). Zinc finger protein transcription factors (e.g., Sp1, Sp3) bind GC box elements in promoters to facilitate transcription [45, 46]. Zn^2^^+^, as a structural cofactor for zinc finger proteins, indirectly regulates transcription factor binding and activation [47].

Double fluorescence staining with a Zn^2^^+^ probe and SIRT3 under TBHP‐induced oxidative stress showed that TBHP caused Zn^2^^+^ loss and inhibited SIRT3 expression. In contrast, exogenous ZnG restored intracellular Zn^2^^+^ puncta and SIRT3 levels, effects that were blocked by the Zn^2^^+^ chelator TPEN (Figure 4D). Molecular docking analysis revealed that SIRT3 residues CYS‐256, CYS‐259, CYS‐280, and CYS‐283 are key Zn^2^^+^ binding sites, forming four ionic bonds with bond lengths of 2.4, 2.2, 4.0, and 2.1 Å, respectively. The binding free energy of this interaction was calculated to be −5.2 kcal/mol, indicating a robust binding affinity between SIRT3 and Zn^2^^+^. This stable protein‐ligand complex formation is critical for maintaining the structural conformation and enzymatic activity of SIRT3 (Figure 4E). Overexpression of SIRT3 (oe‐SIRT3) in NPCs under TBHP stimulation improved mitochondrial morphology and mitigated Δψm decline (Figure S4E). PINK1 and Parkin detection after oe‐SIRT3 revealed activation of mitophagy‐related proteins (Figure S4F). As a mitochondrial deacetylase, SIRT3 regulates energy metabolism and antioxidant responses by deacetylating mitochondrial proteins. SIRT3 activates the SOD2 pathway, enhances mitochondrial ROS scavenging, reduces oxidative stress‐induced mitochondrial damage, and inhibits the accumulation of abnormal mitochondria [48, 49].

SIRT3 serves as a key regulator of intracellular protein acetylation homeostasis. To identify downstream substrates of SIRT3‐mediated deacetylation, we performed co‐immunoprecipitation (Co‐IP) combined with acetylated protein mass spectrometry (MS) in pcDNA‐SIRT3‐overexpressing cells and empty vector controls. Among these candidate interacting proteins, ANXA2 was selected for further investigation. Co‐IP confirmed endogenous interaction, and exogenous co‐transfection of FLAG‐ANXA2 and HA‐SIRT3 further verified binding (Figure 4F,G). SIRT3 knockdown (si‐SIRT3) significantly increased acetylated ANXA2 (Ac‐ANXA2), indicating that SIRT3 is a key deacetylase for ANXA2 (Figure 4H). MS analysis identified K302 and S2 acetylation sites of ANXA2 in the OE‐NC control group. Upon SIRT3 overexpression (OE‐SIRT3), acetylation at K302 was abolished, whereas acetylation at S2 remained unaltered. Notably, acetylation at the S2 site is an irreversible N‐terminal co‐translational modification that is not regulated by SIRT3, confirming that K302 serves as the primary deacetylation target of SIRT3 (Figure 4I). The ANXA2 K302 mutation (K302R; chr15(q22.2)) solely renders this specific site (i.e., lysine 302) refractory to SIRT3‑mediated deacetylation (Figure 4J,L), while K302 is highly conserved across species (Figure 4K). Among different degenerative grades, ANXA2 acetylation was the strongest in Grade V (Figure S5E). We performed cytoplasmic and mitochondrial fractionation (Figure S5G). The results demonstrated that ANXA2 undergoes translocation from the cytoplasm to mitochondria during IVDD, which is consistent with the findings of Zhou et al. [50]. Moreover, fluorescence co‑localization analysis (Figure S5H) further confirmed the colocalization of SIRT3 and ANXA2, providing evidence for the binding of ANXA2 to mitochondrial SIRT3.

### 2.5 Acetylated ANXA2 Tightly Binds mTOR to Inhibit Downstream Autophagy

**FIGURE 5:**
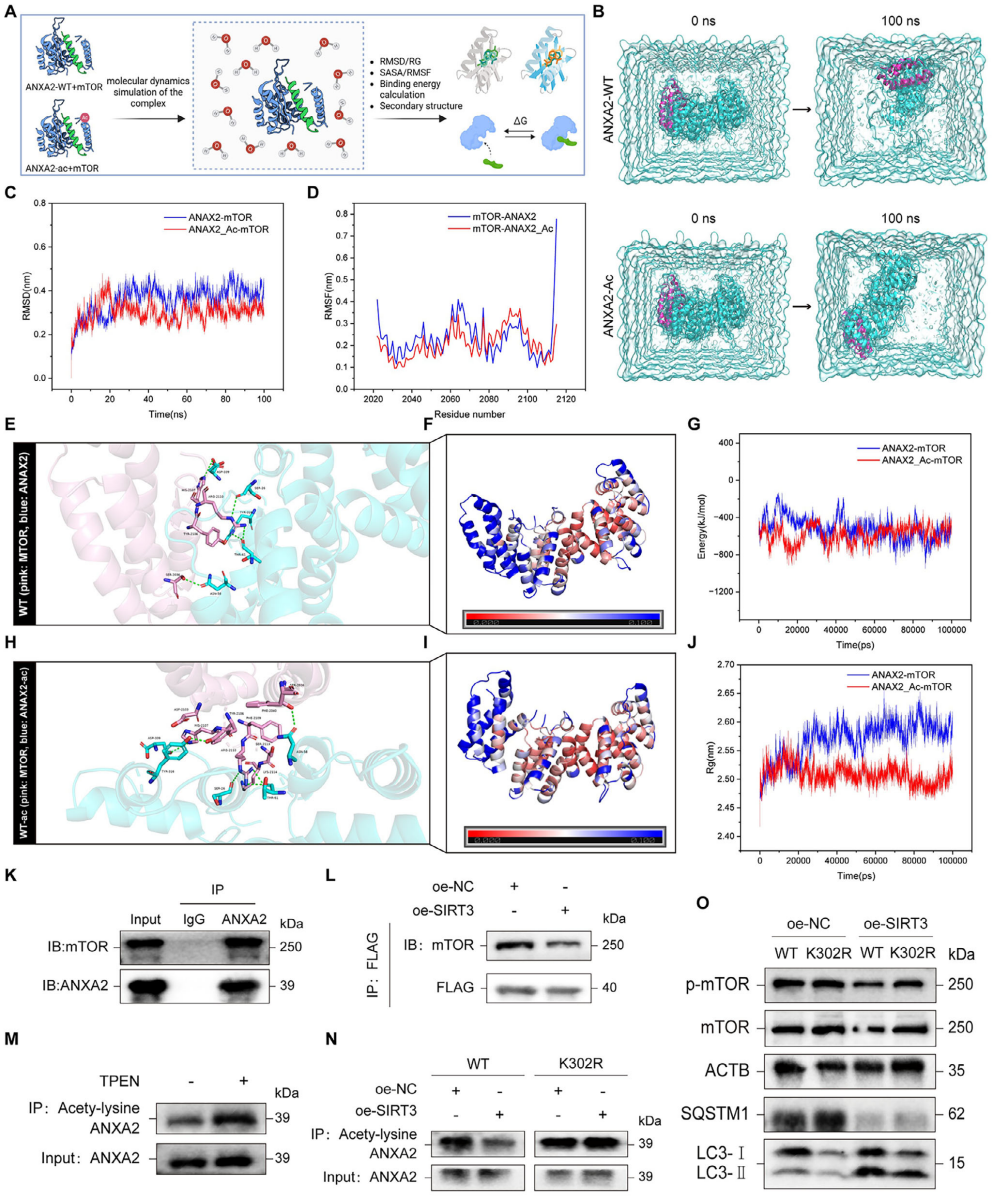
Molecular dynamics simulation and Co‐IP verification of the binding between wild‐type ANXA2, acetylated ANXA2 and mTOR. (A) Schematic diagram of molecular dynamics simulations of ANXA2 WT and ANXA2_Ac bound to mTOR. (B) Conformational overlay of the ANAX2 WT / ANXA2_Ac‐mTOR complex systems after MD simulations (ns and 100 ns). (C) RMSD plots of the ANAX2 WT / ANAX2_Ac‐mTOR complex systems. (D) RMSF plots of mTOR in complexes with ANXA2 WT and ANXA2_Ac. (E) Docking model of wild‐type ANXA2 with mTOR (mTOR: pink; ANXA2: blue; hydrogen bonds: green). (F) B‐factor (RMSF) colored structural diagram (ANAX2‐mTOR). (G) Binding energy analysis reveals better stability of the ANXA2_Ac‐mTOR complex. (H) Docking model of ANXA‐ac with mTOR (mTOR: pink; ANXA‐ac: blue; hydrogen bonds: green). (I) B‐factor (RMSF) colored structural diagram (ANAX2_Ac‐mTOR). (J) Rg analysis shows a more compact structure of the ANXA2_Ac‐mTOR complex. (K) Co‐IP confirms the interaction between mTOR and ANXA2 (n = 3). (L) Detection of the binding ability between FLAG‐ANXA2 and mTOR under SIRT3 overexpression using Co‐IP (n = 3). (M) After TPEN addition, ANXA2 protein was immunoprecipitated from cell lysates, and the acetylation level of endogenous ANXA2 was determined using an anti‐acetylated lysine antibody (n = 3). (N) Detection of the effect of SIRT3 on Ac‐ANXA2 in WT and K302R mutant cells (n = 3). (O) Overexpression of SIRT3 in WT or K302R mutant cells, followed by detection of the listed protein levels via western blot (n = 3).

Our previous work has demonstrated that ANXA2 participates in mitophagy [51], and ANXA2 is reported to modulate mTOR, a core autophagy regulator [52]. SIRT3 regulates ANXA2 acetylation, and differential SIRT3 expression across groups leads to varied acetylation levels of ANXA2 (Figure 4H; Figure S5E). We therefore constructed wild‐type (WT) and acetylated ANXA2 to investigate their binding affinity with mTOR (Figure 5A). Results from 100 ns molecular dynamics simulations showed that both complexes exhibited compact conformations at the initial state. After 100 ns simulation, the N‐terminus and flexible loops of WT ANXA2 obviously shifted outward, while the acetylated mutant maintained a more compact structure with smaller displacement (Figure 5B). Root‐mean‐square deviation (RMSD) analysis revealed that acetylation improved the conformational stability of the complex. After equilibration within 0–20 ns, the WT complex had fluctuating RMSD values ranging from 0.35 to 0.50 nm, whereas the acetylated mutant presented lower and more stable RMSD values (0.25–0.35 nm), indicating tighter ligand binding (Figure 5C). Root‐mean‐square fluctuation (RMSF) results indicated that acetylation reduced the overall flexibility of the complex. The peak RMSF value of the N‐terminus and other flexible regions of ANXA2 decreased from 0.52 to 0.33 nm after acetylation, accompanied by enhanced protein rigidity. Residue fluctuations within the mTOR binding pocket were also markedly suppressed (Figure 5D; Figure S5C). Structural analysis showed that WT ANXA2 bound to mTOR via an extensive hydrogen bond network and contained large flexible regions (Figure 5E,F). By contrast, the ANXA2_Ac mutant relied on combined polar and hydrophobic interactions, resulting in a global rearrangement of intermolecular forces (Figure 5H). The mutant displayed fewer highly flexible regions, more uniform residue fluctuations, and higher structural rigidity (Figure 5I). Acetylation strengthened the receptor‐ligand interaction, minimized dynamic changes at the binding interface, and promoted overall structural stability. Binding energy analysis showed that the WT complex had an average binding energy of −516.14 kJ/mol with a standard deviation of 120.96 kJ/mol. The acetylated complex had a lower average binding energy (−582.41 kJ/mol) and a smaller standard deviation (93.44 kJ/mol), reflecting stronger and more stable binding (Figure 5G). Radius of gyration (Rg) analysis confirmed a more compact structure in the acetylated complex, with Rg values stabilized at 2.48–2.54 nm compared with 2.55–2.62 nm in the WT group (Figure 5J). The two groups had comparable mean SASA values, while the acetylated complex showed reduced SASA fluctuations and more stable surface exposure (Figure S5A). DSSP analysis verified that neither complex underwent large‐scale unfolding during simulation (Figure S5B). This simulation only yields computational experimental results and serves as a reference for subsequent wet‐lab experimental validation.

The fluorescence co‑localization of ANXA2 and mTOR was observed (Figure S5D). The interaction between ANXA2 and mTOR was further verified by Co‐IP assays (Figure 5K). This interaction was weakened upon SIRT3 overexpression‐induced ANXA2 deacetylation (Figure 5L). Given the low zinc microenvironment in pathological conditions, we used TPEN to chelate intracellular Zn^2+^ and assess ANXA2 acetylation. Zn^2+^ depletion by TPEN increased Ac‐ANXA2 levels (Figure 5M) and strengthened the ANXA2‐mTOR interaction in a time‐dependent manner (Figure S5F). SIRT3 overexpression downregulated Ac‐ANXA2 in WT cells but exerted no effect in K302R mutants, verifying the essential role of K302 acetylation (Figure 5N). Functional assays demonstrated that SIRT3‐mediated deacetylation at ANXA2 K302 suppressed mTOR activation, and this regulatory effect was lost in the K302R mutant (Figure 5O). We simultaneously detected mTOR‐related molecules including p‐AMPK/AMPK, p‐mTOR/mTOR, and SLC39A14. Knockdown of SLC39A14 via lentiviral shRNA (LV‐shSLC39A14) followed by ANXA2 overexpression markedly elevated p‐mTOR levels (Figure S5I). We further examined the canonical mTOR downstream targets p‐4EBP1 (Thr37/46) and p‐S6K (Thr389). SIRT3 overexpression inhibited the phosphorylation of 4EBP1 and S6K in WT cells, whereas this inhibitory effect was abrogated in K302R mutants (Figure S5J).

In conclusion, this study identifies a novel SIRT3‐ANXA2/mTOR regulatory complex, in which ANXA2 K302 acts as a core functional regulatory site.

### 2.6 ANXA2‐mTOR Regulates Cellular Senescence by Inhibiting Mitochondrial Function and Autophagy

**FIGURE 6:**
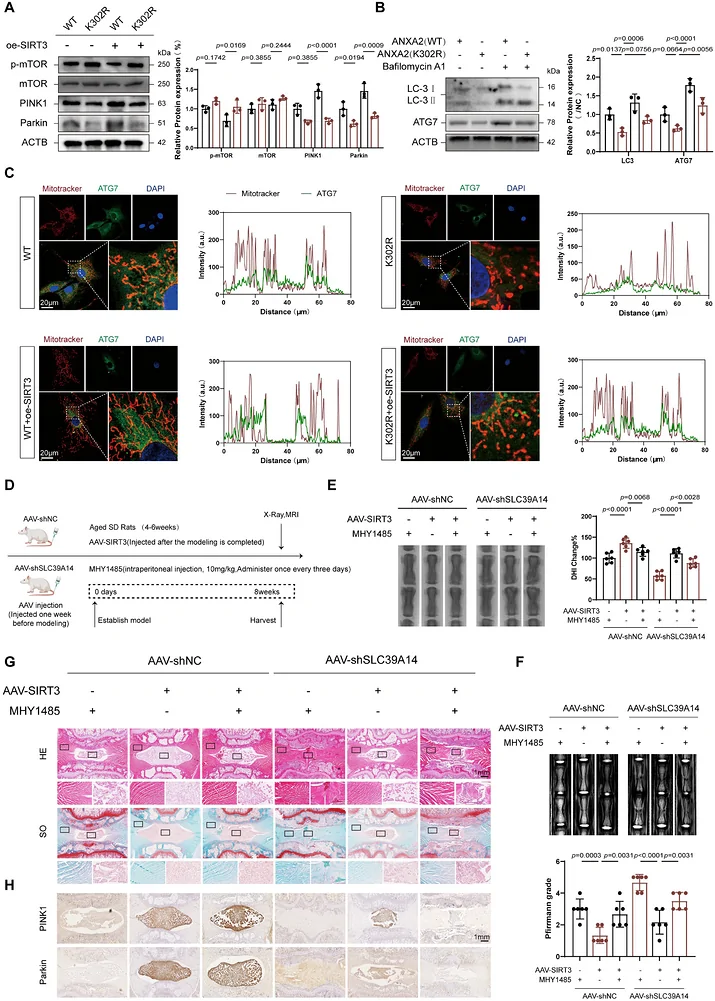
Regulation of mitophagy by the ANXA2‐mTOR complex. (A) Overexpression of SIRT3 in WT and K302R mutant cells, followed by detection of the protein expression of autophagy‐related proteins (p‐mTOR, mTOR, PINK1, Parkin) (n = 3). (B) Addition of Bafilomycin A1(a specific inhibitor that suppresses lysosomal hydrolase activity and autophagosome‐lysosome fusion) to ANXA2 WT and ANXA2 K302R cells, followed by detection of the protein levels of autophagic proteins (LC3, ATG7, P62) (n = 3). (C) Double fluorescence staining with MitoTracker and ATG7 to detect mitochondrial morphology and autophagic flux (n = 6; Scale bar: 20 µm). (D) Schematic diagram of the injection and modeling strategy for adeno‐associated virus (AAV)‐shSLC39A14, AAV‐SIRT3, and MHY1485. (E) X‐ray images showing the height of caudal intervertebral spaces in rats after different treatments (n = 6). (F) MRI images showing the structural integrity and water content of NP tissue in rat caudal intervertebral discs following different interventions (n = 6). (G) HE and SO staining images of the caudal intervertebral disc in rats after different treatments (n = 6; scale bar: 1 mm for HE and SO staining). (H) IHC staining for the expression levels of PINK1 and Parkin in rat tissue (n = 6; Scale bar = 1 mm). Note: mitophagy flux (LC3II/I + Baf A1); static marker levels (Mitotracker Red, PINK1, Parkin).

To verify the regulatory effects of ANXA2 K302 acetylation on the mTOR pathway and autophagy, we overexpressed SIRT3 in WT and ANXA2 K302R mutant cells. Western blot was first used to detect the expression of phosphorylated mTOR (p‐mTOR), total mTOR, PINK1, and Parkin for evaluating basal pathway activity. The results showed that ANXA2 bound to mTOR in WT cells and promoted mTOR phosphorylation and activation. Activated mTOR suppressed the PINK1‐Parkin pathway, while SIRT3 overexpression partially reduced p‐mTOR levels and relieved the inhibition of PINK1‐Parkin‐mediated autophagy (Figure 6A). Meanwhile, SIRT3 overexpression promotes the nuclear translocation of TFEB, whereas the ANXA2 K302R mutant abolishes this effect (Figure S6A). To distinguish between static autophagy levels and dynamic turnover, we performed an autophagic flux assay. We used Bafilomycin A1 (a specific inhibitor that suppresses lysosomal hydrolase activity and autophagosome‐lysosome fusion) to block the fusion of autophagosomes with lysosomes and inhibit autophagy, thereby evaluating the net autophagosome formation capacity. WT cells exhibited a relatively higher autophagosome formation capacity than K302R mutant cells (Figure 6B). We further monitored the recruitment of autophagy machinery to mitochondria in WT and K302R mutant cells using MitoTracker staining combined with double fluorescence staining of ATG7 and LC3 (Figure 6C; Figure S6C). Regarding the effect of the ANXA2 mutant on intracellular mitochondrial morphology and autophagy, the results demonstrated that SIRT3 expression helped preserve mitochondrial morphological integrity and autophagic flux. However, when the K302 site of ANXA2 was mutated, the ANXA2‐mediated inhibition of mitophagy could not be rescued by SIRT3 overexpression (Figure 6C). In addition, static evaluation of mitochondrial health was performed: JC‐1 staining (Figure S6D), TEM (Figure S6E), and MitoSOX staining (Figure S6B) collectively confirmed the critical role of the ANXA2 K302 site in the rescue of mitochondrial morphology and function by SIRT3. Mitochondria are essential organelles for cellular function, and mitochondrial damage is closely associated with cellular senescence [53]. We evaluated the senescence level and SASP secretion in WT and K302R mutant cells via western blot analysis. Intracellular levels of TP53, CDKN1A, and CDKN2A were increased in K302R cells (Figure S6F), accompanied by elevated secretion of SASP‐related proteins (TNF‐α, IL‐1β, IL‐16) and an increased number of SA‐β‐gal‐positive cells (Figure S6G). SIRT3 overexpression partially alleviated SASP secretion; however, it exerted only a weak ameliorative effect on K302R cells.

We further investigated the in vivo efficacy of SIRT3 expression in regulating ANXA2 and activating the mTOR pathway. Rats were divided into two groups and treated with AAV‐shNC or AAV‐shSLC39A14. An AAV injection was administered 1 week before the official experiment; after 1 week of feeding, a needle puncture‐induced IVDD model was established. In the AAV‐shNC and AAV‐shSLC39A14 groups, AAV‐SIRT3 was administered and MHY1485 (an in vivo mTOR agonist) was injected intraperitoneally, respectively. After 8 weeks, X‐ray and MRI scans were performed on the experimental animals (Figure 6D). DHI results for intervertebral disc height revealed that maintaining SIRT3 expression in vivo preserved intervertebral space height. In contrast, MHY1485 reduced intervertebral space height by inhibiting autophagy, and this effect was more pronounced after SLC39A14 channel silencing (Figure 6E). T2‐weighted MRI images showed high signal intensity in NP tissue; furthermore, high SIRT3 expression partially rescued the autophagy inhibition induced by mTOR activation (Figure 6F). HE and SO staining of NP tissue sections revealed that SLC39A14 channel activity plays a critical role in maintaining NP integrity. SIRT3 expression contributed to the preservation of NP morphology and integrity, while partially alleviating the damaging effects of mTOR activation on NPCs (Figure 6G).

Immunohistochemical staining of ANXA2, mTOR, PINK1, and Parkin was performed on tissue sections. PINK1‐Parkin is essential for maintaining NP tissue. Meanwhile, in tissues with SLC39A14 knockdown, the expression levels of mTOR and ANXA2 were upregulated, corresponding to PINK1‐Parkin autophagy inhibition and impaired NP integrity (Figure 6H; Figure S7A). Histological scores were visualized in the form of a heatmap (Figure S7B), while semi‐quantitative analysis of the immunohistochemical results is shown (Figure S7C). Notably, AAV‐SIRT3 alone or with MHY1485 minimally altered PINK1/Parkin in AAV‐shNC animals. In contrast, PINK1/Parkin were markedly reduced in AAV‐shSLC39A14 animals, with differences between the two SIRT3‐treated groups. Combined with prior reports of SIRT3 activating PINK1/Parkin via alternative pathways [54, 55], our data demonstrate that SIRT3's regulation of PINK1/Parkin in vivo is dependent on SLC39A14.

### 2.7 Sustained Zn^2^^+^ Influx Modulates Autophagic Flux via the ANXA2‐mTOR Complex and Facilitates the Degradation of ANXA2

**FIGURE 7:**
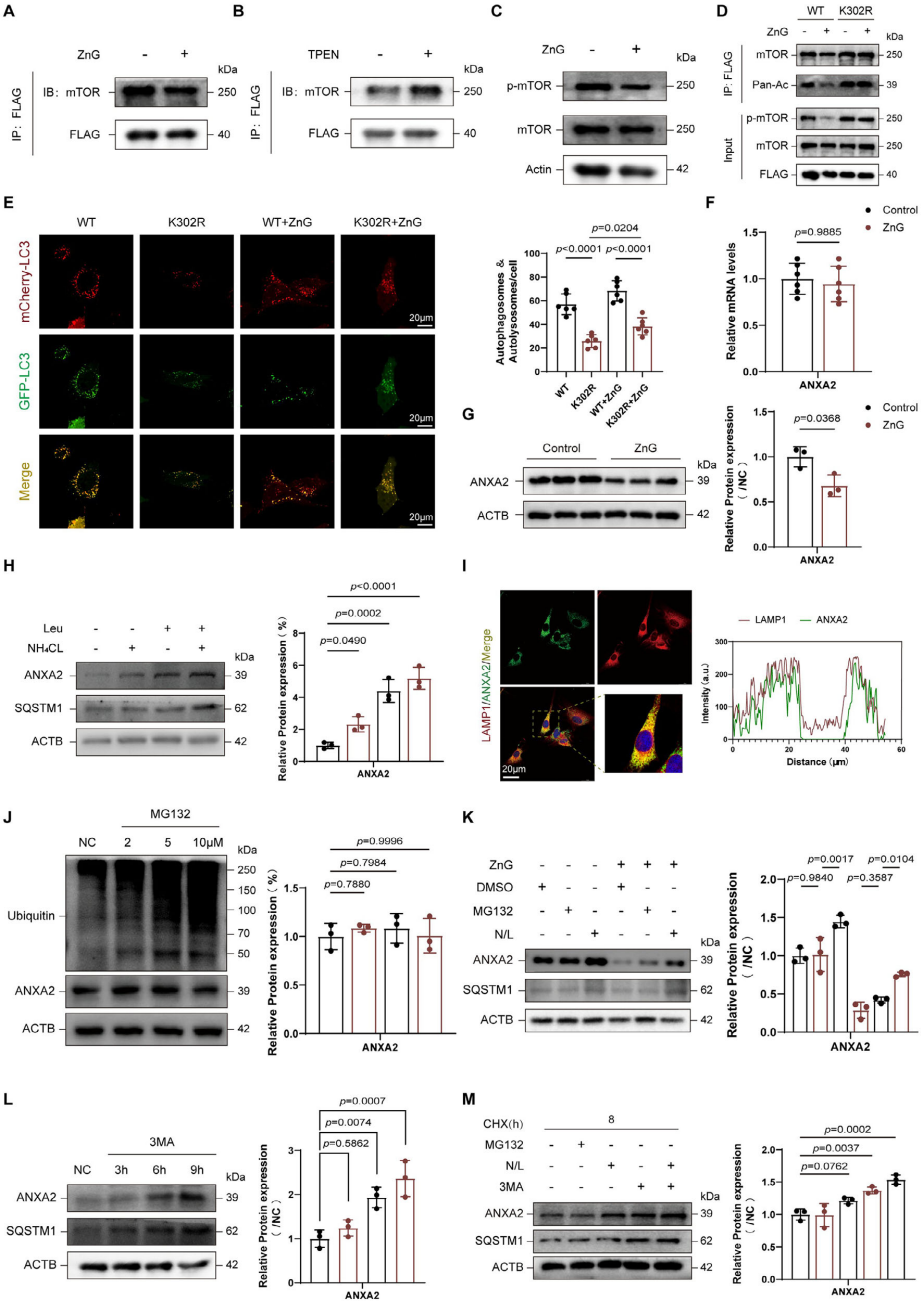
Zn^2^^+^ regulates the degradation of ANXA2 via the autophagy‐lysosomal pathway. (A) Comparison of the binding capacity between ANXA2‐FLAG and mTOR before and after ZnG treatment (n = 3). (B) Detection of the binding ability between FLAG‐ANXA2 and mTOR under TPEN treatment using Co‐IP (n = 3). (C) WB analysis of the differences in p‐mTOR and mTOR levels before and after ZnG treatment (n = 3). (D) Detection of mTOR bound to ANXA2‐FLAG, total protein acetylation, and p‐mTOR/mTOR in input samples in WT and K302R cells following ZnG treatment (n = 3). (E) Evaluation of autophagy alterations under different ZnG concentrations using mCherry‐LC3/GFP‐LC3 dual‐labeled lentivirus (n = 6, Scale bar: 20 µm). (F) ANXA2 mRNA levels before and after ZnG treatment (n = 6). (G) ANXA2 protein levels before and after ZnG treatment (n = 3). (H) ANXA2 protein levels after lysosomal inhibition with Leupeptin (Leu, a serine and cysteine protease inhibitor) and NH_4_Cl (a lysosomal acidification inhibitor) (n = 3). (I) Immunofluorescence staining of the lysosomal marker LAMP1 and ANXA2 (n = 6; Scale bar: 20 µm). (J) WB analysis of ANXA2 after treatment with different concentrations of MG132(a highly efficient and specific proteasome inhibitor), with Ubiquitin used as a positive control (n = 3). (K) Detection of ANXA2 protein levels after different treatments (ZnG, DMSO, MG132, N/L [Leupeptin + NH_4_Cl]) (n = 3). (L) ANXA2 protein levels after autophagy blockage with the 3‐methyladenine (3‐MA) (n = 3). (M) Identification of the ANXA2 protein degradation pathway via CHX (a protein synthesis inhibitor)‐mediated protein synthesis inhibition, combined with subsequent treatment with MG132, N/L, or 3‐MA (n = 3).

We found that Zn^2^^+^ influx mediated by the SLC39A14 channel in healthy NPCs effectively maintains the function of SIRT3 in both cells and mitochondria. We also confirmed that the inhibitory effect of SIRT3 on ANXA2‐mTOR‐mediated autophagy depends on the K302 site of ANXA2 and Zn^2+^. Accordingly, we examined the effect of ZnG addition on the ANXA2‐mTOR complex. The results showed that the presence of Zn^2^^+^ was unfavorable for the formation of the ANXA2‐mTOR complex (Figure 7A,B). Furthermore, ZnG impaired mTOR phosphorylation (Figure 7C). To further investigate this phenomenon, we modulated intracellular Zn^2^^+^ concentration in cells expressing WT ANXA2 and ANXA2 with the K302R mutation. Zn^2^^+^ regulated the binding between ANXA2 and mTOR, the acetylation status of ANXA2, and the activation of p‐mTOR in cells expressing WT ANXA2. However, Zn^2^^+^ had no significant effect in cells expressing the K302R mutant (Figure 7D). Given that mTOR serves as an upstream inhibitory factor of autophagy, we re‐evaluated intracellular autophagic activity using mCherry‐LC3 and GFP‐LC3. The results of autophagy detection were consistent with the activation status of p‐mTOR (Figure 7E), confirming the regulatory role of Zn^2^^+^ in the autophagy inhibitory switch.

We evaluated ANXA2 expression at both the mRNA and protein levels in the ZnG and control groups. Exogenous Zn^2^^+^ supplementation had no significant effect on ANXA2 transcription, but it influenced ANXA2 protein content (Figure 7F,G). This indicates that Zn^2^^+^ regulates ANXA2 post‐transcriptionally or at the level of protein metabolism. Next, we explored the intracellular degradation pathways of ANXA2, which mainly include the lysosomal pathway and the ubiquitin‐proteasome pathway. First, to determine whether ANXA2 is degraded via the lysosomal pathway, we used Leupeptin (Leu, a serine and cysteine protease inhibitor) and NH_4_Cl (a lysosomal acidification inhibitor) to inhibit lysosomal function. Both Leu and NH_4_Cl treatment inhibited ANXA2 degradation (Figure 7H), and their combination (N/L) maintained ANXA2 levels in a time‐dependent manner (Figure S8A). Subsequently, we detected the expression of ANXA2 and the lysosomal outer membrane protein Lysosomal Associated Membrane Protein 1 in cells; immunofluorescence results showed partial colocalization of these proteins (Figure 7I). In addition, we used Z‐Leu‐Leu‐Leu‐al (MG132, a highly efficient and specific proteasome inhibitor) to inhibit the ubiquitin‐proteasome pathway, observing no significant change in ANXA2 levels (Figure 7J).

We further detected the effects of N/L and MG132 on ANXA2 degradation in cells with or without exogenous Zn^2^^+^ supplementation. Zn^2^^+^ partially reduced the accumulation of ANXA2 caused by N/L‐induced inhibition (Figure 7K). We simultaneously added N/L to cells with or without SIRT3 overexpression to detect ANXA2 retention. Western blot results showed that SIRT3 promoted partial degradation of ANXA2, while N/L treatment blocked the lysosomal pathway, leading to partial retention of ANXA2 (Figure S8B). Subsequently, we used the autophagy inhibitor 3‐methyladenine (3‐MA) and the autophagy activator rapamycin to inhibit or activate autophagy, respectively. ANXA2 expression was retained with autophagy inhibition and decreased with autophagy activation (Figure 7L; Figure S8C). Next, we added CQ as a lysosomal inhibitor, which led to ANXA2 accumulation (Figure S8D). We then used CQ followed by Cycloheximide (CHX, a protein synthesis inhibitor) to suppress protein synthesis and analyzed samples at 0, 2, 4, and 8 h. After CHX‐mediated inhibition of protein synthesis, ANXA2 content in the control group decreased over time, whereas ANXA2 content in the CQ group showed no significant time‐dependent decrease (Figure S8E). Finally, we used CHX and added MG132, N/L, or 3‐MA to detect the effect of autophagy on ANXA2. Inhibition of both the autophagic and lysosomal pathways led to ANXA2 accumulation (Figure 7M).

Integrating previous results, we found that ANXA2 undergoes deacetylation under sufficient intracellular Zn^2^^+^ levels. This deacetylated state weakens the binding between ANXA2 and mTOR, relieving mTOR's inhibitory effect on autophagy and promoting autophagy activation. This explains the maintenance of normal autophagic flux in healthy cells, and efficient autophagic flux further reduces intracellular ANXA2 levels.

In senescent cells, inactivation of SLC39A14 and the resultant reduction in Zn^2^^+^ levels lead to enhanced acetylation of ANXA2. This hyperacetylated ANXA2 exhibits stronger binding to mTOR, which strengthens autophagy inhibition. This suppressed autophagy prevents ANXA2 degradation, resulting in ANXA2 accumulation and the formation of a deleterious intracellular feedback loop.

## 3 Discussion

IVDD is widely recognized as an important factor underlying chronic low back pain in middle‐aged and older adults. Its development is associated with inflammatory responses, mechanical loading, oxidative damage, and metabolic dysfunction, yet the underlying mechanisms remain incompletely elucidated [56, 57]. Cellular senescence is now acknowledged as a central pathogenic driver of IVDD. Senescent nucleus pulposus cells secrete abundant pro‐inflammatory, catabolic factors within the intervertebral disc microenvironment, accelerating extracellular matrix degradation and tissue degeneration [58]. Senescence and collapse of NPCs induce mechanical changes in the surrounding core region and the longitudinal spine, which are particularly critical for the initiation and progression of IVDD [59]. Currently, clinical interventions focus on nonsteroidal anti‐inflammatory drugs (NSAIDs) to alleviate pain symptoms and surgical resection of herniated degenerated NP tissue. These approaches fail to fundamentally address the challenge of delaying NPC senescence [60, 61]. Novel molecular targets and regulatory pathways that can counteract NPC senescence remain to be urgently identified.

**FIGURE 8:**
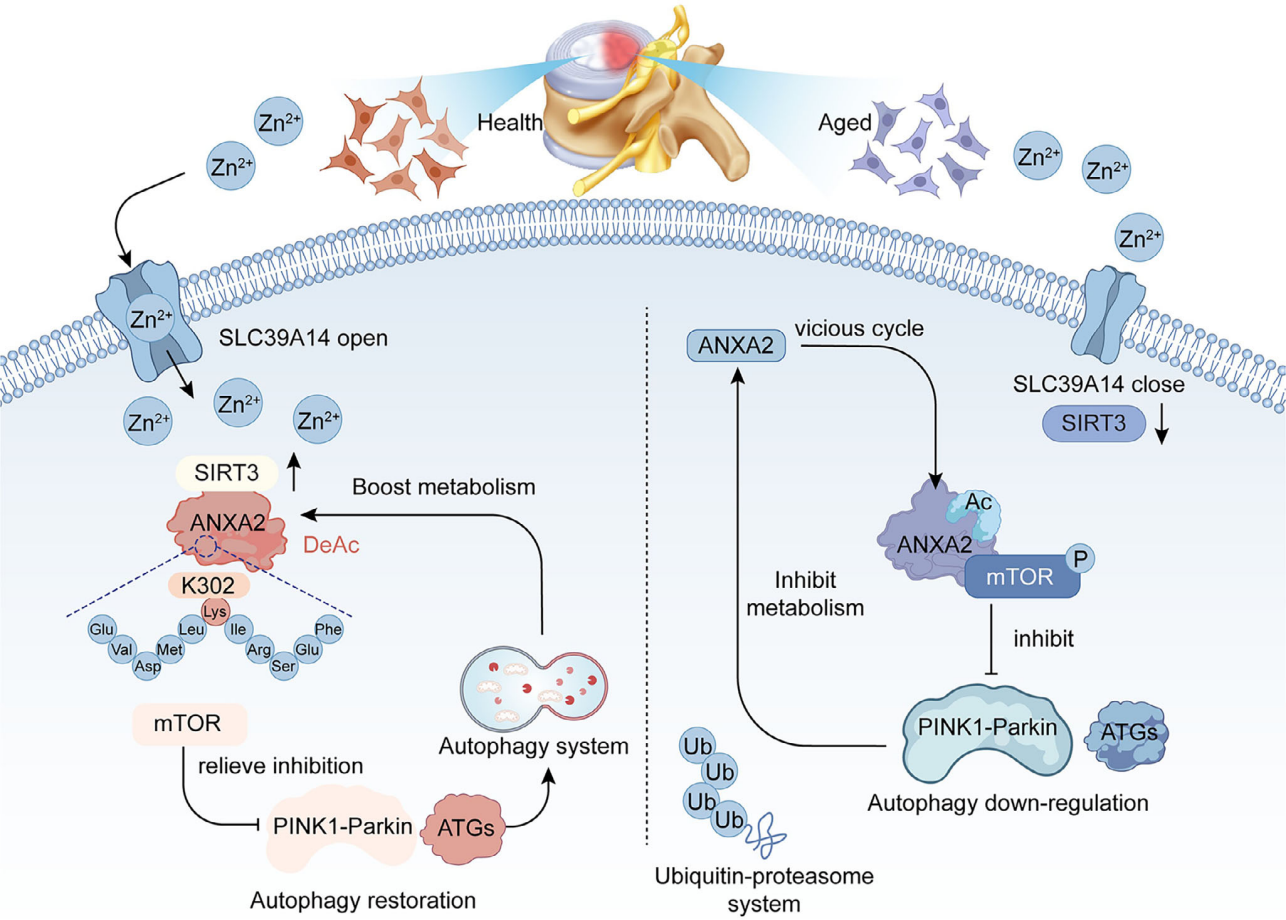
The mechanism of the “Zn2+‐autophagic flux” regulating ANXA2 autometabolism and affecting mitophagy in intervertebral disc degeneration.

The core innovation of this study lies in the identification and definition of the “Zn^2^^+^‐autophagic flux” (Figure 8): an intracellular regulatory node centered on the K302 residue of ANXA2. This regulatory mode integrates Zn^2^^+^ signaling and autophagic flux to regulate NPC senescence. To our knowledge, this is the first report of a Zn^2^^+^‐dependent, site‐specific regulatory module governing autophagic flux in NPCs associated with IVDD. This regulatory system exerts its function via the following molecular cascade: upstream, the membrane‐localized zinc transporter SLC39A14 mediates Zn^2^^+^ influx. High SLC39A14 expression in healthy NPCs maintains sufficient intracellular Zn^2^^+^ as an “activation signal”; Zn^2^^+^ then activates mitochondrial deacetylase SIRT3 [49, 62]. Activated SIRT3 targets ANXA2 and deacetylates its K302 residue. This modification changes the conformation of the ANXA2‐mTOR complex, weakens intermolecular interaction between the two proteins [63], decreases mTOR phosphorylation, and ultimately alleviates mTOR‐mediated suppression of autophagic flux [64]. When SLC39A14 is downregulated, intracellular Zn^2^^+^ decreases, SIRT3 activity weakens, and ANXA2 K302 remains persistently acetylated. This enhances ANXA2‐mTOR binding, exacerbates the suppression of autophagic flux, and blocks autophagy‐lysosomal degradation of ANXA2. This switch differs from previously identified mitophagy regulators (e.g., PINK1‐Parkin [65], NIX/BNIP3 [66]) in two core aspects: first, it defines a novel Zn^2^^+^‐dependent homeostasis mechanism; second, it acts via site‐specific deacetylation of ANXA2. Moreover, the evolutionary conservation of the K302 residue across species suggests it may represent a conserved regulatory mechanism beyond IVDD, with broader biological significance.

Our previous studies have demonstrated that dysregulated autophagic flux and aberrant epigenetic modifications in senescent NPCs are key risk factors driving IVDD progression [51, 67]. Targeted modulation of critical molecules to restore autophagic flux has been shown to mitigate mitochondrial dysfunction and ultimately delay IVDD progression in preliminary in vitro and in vivo models [68]. However, the upstream mechanisms regulating autophagic flux dysregulation remain insufficiently characterized, particularly the synergistic interaction between metal ion signaling and post‐translational protein modifications, as well as their specific association with NPC senescence in the context of IVDD.

In this study, senescent NPCs showed reduced intracellular Zn^2^^+^ and decreased LC3‐II levels compared with young NPCs. Immunofluorescence co‐staining of LC3 and MitoTracker further revealed that inhibition of Zn^2^^+^ transport channels impaired autophagosome formation in the mitochondrial compartment, indicating that Zn^2^^+^ signaling and zinc transport channels regulate autophagosome formation during IVDD. We next investigated the underlying mechanism. SLC39A14, a plasma membrane‐localized zinc transporter, is essential for maintaining intracellular Zn^2^^+^ homeostasis. However, its role in autophagic flux dysregulation and NPC senescence in IVDD has been largely unexplored. We found that both SLC39A14 protein and mRNA were significantly downregulated in senescent NPCs. Consistently, expression changes in downstream molecules (SIRT3, ANXA2, and mTOR) correlated with SLC39A14, supporting a regulation model via SLC39A14‐mediated Zn^2^^+^ signaling. Functionally, SLC39A14 silencing reduced LC3‐II and ATG5, inhibited autophagosome formation, and suppressed autophagic flux, confirming that dysregulated Zn^2^^+^ signaling mediated by SLC39A14 drives autophagic impairment and NPC senescence in IVDD.

As a major post‐translational modification, protein acetylation regulates protein function and intracellular signaling by modifying specific lysine residues, thereby altering protein charge, spatial conformation, and the capacity for intermolecular interactions [69]. Notably, acetylation‐driven functional alterations of ANXA2 endow it with novel regulatory functions, rendering it a critical node in this process [70].

At present, no FDA‐approved drugs are available for IVDD treatment that target Zn^2^^+^ regulation, but some existing drugs may have repurposing potential. Oral Zn^2^^+^ supplements, such as zinc gluconate, can increase systemic Zn^2^^+^ levels [71], and for patients with advanced IVDD, injectable Zn^2^^+^ formulations can be delivered locally. Notably, several small‐molecule acetylation inhibitors already show therapeutic effects on hyperacetylation‐related diseases. For example, Remodelin modulates mitochondrial lipid metabolism in cancer cells by inhibiting N‐acetyltransferase 10 (NAT10) [72], while A‐485 improves mitochondrial function in osteoblasts by promoting deacetylation of glutamate dehydrogenase 1 (GLUD1) [73]. In IVDD treatment, either repurposing approved drugs or developing novel agents that target specific acetyl groups to regulate epigenetic modifications and restore autophagic flux represents an ideal strategy for enhancing cell viability and mitochondrial function. This approach provides a foundation for clinical intervention at the current stage.

## 4 Limitations of the Study

This study uncovers a novel molecular mechanism by which the SLC39A14/Zn^2^^+^/SIRT3/ANXA2 signaling axis regulates autophagic flux and nucleus pulposus cell senescence during intervertebral disc degeneration. Several limitations of this work are presented below. First, only male clinical specimens and experimental animals were recruited to avoid the disturbance of estrogen on systemic ionic homeostasis. This setting restricts the generalizability of our results to female individuals, and future studies incorporating subjects of both sexes are needed for verification. Second, strictly age‐matched surgical patients with healthy intervertebral discs could not be enrolled owing to practical clinical limitations, resulting in potential age bias. Intra‐group comparisons within degenerative cohorts were therefore predominantly adopted to alleviate the influence of this confounding factor. Third, MHY1485 used in animal experiments exhibits dual biological functions: it activates the mTORC1 pathway and inhibits autophagosome‐lysosome fusion via an mTOR‐independent route. These dual effects may cause confounding interference and hinder the interpretation of in vivo data; targeted genetic intervention is required in subsequent studies to confirm our findings. Lastly, the dissociation kinetic analyses in this work are merely computational simulations, which only act as auxiliary references for further in vitro and in vivo functional experiments rather than conclusive biological evidence.

## 5 Materials and Methods

### 5.1 Human Samples

All human NP samples in this study were obtained from surgical patients at the Second Affiliated Hospital of Wenzhou Medical University. The protocol was approved by the hospital Medical Ethics Committee (Ethics Approval Number: 2024636). The IVDD group included 60 male patients (age 42–75) who underwent discectomy for lumbar disc herniation. Preoperative lumbar T2WI assessed IVDD severity. All MRI images were independently evaluated twice by three experienced spinal surgeons blinded to the study information, with a 4‐week interval between the two evaluation rounds to minimize memory bias. NP samples classified as Pfirrmann Grade II‐V were selected. Patients complicated with liver dysfunction, renal insufficiency, knee osteoarthritis, spinal tumors, infections, trauma, or autoimmune diseases were excluded. The control group comprised 12 male patients (age 25–30) who underwent surgery for thoracolumbar fractures or idiopathic scoliosis; preoperative MRI confirmed no obvious IVDD (Pfirrmann Grade I), and non‐degenerated NP tissues were collected from non‐lesioned areas. Individuals with liver disorders, renal dysfunction, osteoarthritis, prior lumbar surgery, or chronic low back pain were also excluded.

IVDD subgroups were divided into four Pfirrmann grades (II, III, IV, V; 15 patients each) and further consolidated into two analysis groups: Mid‐IVDD (II‐III, 30 patients total) and Severe‐IVDD (IV‐V, 30 patients total). Baseline characteristics (age, gender, height, body weight, BMI, SBP/DBP, and WC) are shown in Figure 1D,E, reported as mean ± standard deviation.

### 5.2 Animal Models

Sprague‐Dawley (SD) rats were used, and the protocol was approved by the Laboratory Animal Ethics Committee of Wenzhou Institute, University of Chinese Academy of Sciences (Ethics No.: WIUCAS25090201). SPF‐grade adult male SD rats (250‐300 g) were selected to avoid sex hormone‐induced variability and minimize confounding factors. Rats were randomly divided into control and puncture model groups (n = 6/group) and fed different diets.

Rats were anesthetized via intraperitoneal injection of 3% (w/v) pentobarbital solution at a dose of 2 mL/kg and fixed in a prone position on a Siemens X‐ray fluoroscope (Germany). A single needle insertion was performed for each target intervertebral disc via percutaneous puncture. The seventh/eighth caudal intervertebral disc was localized, and a sterile hollow 22‐G needle was vertically inserted 3 mm into the NP under real‐time X‐ray guidance. A custom‐made stopper mounted on the needle ensured a consistent insertion depth of 3 mm. After insertion, the needle was rotated 360° and kept in place for 30 s. The needle puncture itself damaged the structure of the nucleus pulposus, and aspiration would lead to excessive tissue destruction; therefore, aspiration was not carried out. Postoperatively, penicillin sodium (200 000 U/kg) was subcutaneously injected daily for 3 consecutive days to prevent infection. For the aged model, 2‐month‐old and 24‐month‐old SPF‐grade male SD rats were randomly assigned to regular zinc (25 mg/kg zinc, standard diet) or high‐zinc (120 mg/kg zinc, zinc gluconate‐supplemented customized diet) groups (n = 6/group). The customized diet matched the standard diet except for zinc content. Rats were fed twice weekly, housed under standard conditions with free access to food and water for 4 months, and body weight was recorded weekly. Intervertebral disc tissues were collected post‐intervention. For AAV‐mediated intervention subgroups (AAV‐shNC and AAV‐shSLC39A14), corresponding regular or high‐zinc diets were initiated 1 week before AAV injection.

### 5.3 Isolation and In Vitro Culture of NPCs

Fresh human NP tissues were instantly placed in serum‐free DMEM/F12 medium with 1% penicillin‐streptomycin (Gibco, 15140148) and transported on ice to the lab within 1 h. Inside a biosafety cabinet, tissues were washed thrice with pre‐cooled PBS containing 1% antibiotics to eliminate blood, annulus fibrosus residues, and contaminants, then minced into ∼1 mm^3^ pieces in sterile dishes and collected in 15 mL tubes. Tissues were digested for 8 h at 37°C under 5% CO_2_ using serum‐free DMEM/F12 supplemented with 0.2 mg/mL type II collagenase (BioFroxx, 2275GR001); tubes were inverted gently every 2 h to improve digestion efficiency. After digestion, the cell suspension was filtered via a 70 µm cell strainer and centrifuged at 300 × g for 5 min at room temperature. The supernatant was discarded, and cell precipitates were resuspended in complete DMEM/F12 medium containing 10% fetal bovine serum (Gibco) and 1% penicillin‐streptomycin. Gentle pipetting was performed to generate single‐cell suspensions. Cells were seeded into T25 flasks for primary culture at 37°C under 5% CO_2_ with saturated humidity. Primary cells at 80–90% confluency were digested and passaged with 0.25% trypsin‐EDTA (Gibco). Stable P2‐generation NPCs were used for subsequent experiments. Consistent with our aim of maintaining intracellular Zn^2^^+^ homeostasis to delay IVDD progression, cells were pretreated with 30 µm ZnG for 24 h before induction of cellular senescence or oxidative stress.

### 5.4 β‐Galactosidase Staining

Cellular senescence of nucleus pulposus cells was measured with β‐galactosidase staining kit (Beyotime, Shanghai). Logarithmically growing NPCs were seeded in 6‐well plates at a density of 5 × 10^4^ cells per well. Culture medium was aspirated, and cells were washed three times (5 min per time) with precooled PBS (pH 7.4). Each well was incubated with 1 mL staining fixative at room temperature (25 ± 2°C) for 15 min. The staining working solution was freshly prepared at a ratio of staining solution: staining buffer: substrate solution = 10:100:1, and 1 mL was added to each well. Cells were incubated overnight in a 37°C CO_2_‐free incubator for 12–16 h in the dark. After incubation, the working solution was removed, and cells were rinsed twice with PBS. Images were acquired under an Olympus BX53 light microscope (Tokyo, Japan). ImageJ software was used to count β‐galactosidase‐positive senescent cells and total cells.

### 5.5 Western Blot Analysis

After discarding the culture medium, logarithmic‐phase cells were washed 2–3 times using precooled PBS (pH 7.4) to remove residual medium and serum. RIPA lysis buffer (Thermo Fisher Scientific, 89901) supplemented with protease‐phosphatase inhibitor cocktail (Beyotime, P1049) and phenylmethylsulfonyl fluoride (PMSF, Beyotime, ST506) was added to culture dishes. Cells were lysed on ice for 30 min with gentle shaking every 5 min to ensure complete lysis. Lysates were collected into pre‐cooled 1.5 mL centrifuge tubes after cell scraping. Centrifuge the mixture at 12,000 × g and 4°C over 30 min. Retain supernatants as total protein specimens and measure protein levels using Beyotime BCA reagent. Run protein samples on 8–12% SDS‐PAGE gels and transfer to PVDF blots. Rapid blocking buffer (Beyotime) is applied, then blots react with primary antibodies at 4°C overnight. Post‐wash incubation with secondary antibodies is conducted, and protein bands are detected by Vazyme ECL chemiluminescence reagent. Details of the antibodies used are provided in the supplementary materials (Table S3).

### 5.6 Reverse Transcription Quantitative Polymerase Chain Reaction (Rt‐qPCR)

Real‐time quantitative polymerase chain reaction was utilized to quantify transcript abundance of target genes. The TB Green Premix Ex Taq II kit (TAKARA, Cat. No. RR037A) was applied for amplification on Bio‐Rad's CFX96 Touch detection device. The 2^−^ΔΔCt approach was used to compute relative mRNA levels, normalized to the internal controls glyceraldehyde‐3‐phosphate dehydrogenase (GAPDH) and β‐actin. Detailed primer information can be found in Table S4. All reactions were run in three technical replicates, and the experiment was validated through three independent biological replicates to ensure the reproducibility of the results.

### 5.7 Immunofluorescence Staining

Seed cells on sterile coverslips (12‐well plates) or confocal dishes. Upon 70%‐80% cell coverage, remove medium and wash cells 3 × 5 min with cold PBS (pH 7.4). 4% PFA (400 µL/well) fixed cells for 15 min at room temperature to preserve morphology, then PBS washed away residual fixative. Permeabilization was achieved via 0.1% Triton X‐100 PBS (10 min, RT), followed by 1‐h blocking with 5% BSA‐PBS (500 µL per well) to suppress non‐specific binding.

Once the blocking step finished, diluted primary antibodies (1:200 in 5% BSA‐PBS) were applied to cells and kept at 4°C overnight. Excess unbound primary antibodies were washed off via three 5‐min PBS rinses the next morning. Cells were then treated with 1:500 diluted Alexa Fluor 488/594 secondary antibodies (Invitrogen) and incubated in darkness at room temperature for 60 min. Three 10‐min PBS washes removed free secondary antibodies. Nucleus staining was performed with 1 µg/mL DAPI (Solarbio, C0065) for 5 min, followed by two PBS washes. Image acquisition was carried out using either an Olympus BX53 fluorescence microscope.

### 5.8 Mitotracker

To mark mitochondria selectively in NPCs, MitoTracker Red CMXRos dye (Cat. M7512, Invitrogen, Waltham, MA) was adopted. A density of 5×10^4^ NPCs was seeded into each well of 24‐well plates, followed by incubation at 37°C with 5% CO_2_ until cells adhered firmly to the plate bottom. After discarding the original medium, cells were gently rinsed twice with pre‐warmed (37°C) HBSS (pH 7.4) to eliminate residual serum. Prepare serum‐free DMEM/F12 mixed with 50 nM MitoTracker Red CMXRos and add the mixture to every well. The culture plates were kept in darkness for a 30‐min incubation at 37°C and 5% CO_2_.

Subsequently, the staining solution was discarded, and cells were washed three times (5 min each) with pre‐cooled HBSS to remove unbound probes. Fresh complete medium (1 mL/well) was added, and imaging was performed immediately using a Zeiss LSM 880 laser scanning confocal microscope (Jena, Germany). Mitochondrial fluorescence signals were quantified via ImageJ software (Version 1.8.0), with mean fluorescence intensity reflecting mitochondrial content and distribution. At least six independent fields of view were analyzed per group.

### 5.9 MitoSOX

Mitochondrial superoxide levels in NPCs were specifically detected using MitoSOX Red (Yeasen Biotech, Shanghai, China, 40778ES50). Briefly, MitoSOX Red powder was dissolved in anhydrous DMSO to prepare a 5 mmol/L stock solution, which was diluted to 5 µmol/L with serum‐free DMEM/F12 immediately before use. NPCs were seeded into 12‐well plates and cultured until fully adherent. After medium removal, cells were gently rinsed twice with pre‐warmed (37°C) HBSS (pH 7.4) to eliminate residual serum. Each well was added with 1 mL serum‐free medium containing 5 µmol/L MitoSOX Red, and plates were incubated in the dark at 37°C with 5% CO_2_ for 10 min. Subsequently, the staining solution was discarded rapidly, and cells were washed three times (5 min each) with pre‐cooled (4°C) HBSS to terminate the reaction and remove unbound probes. Replace the medium with fresh complete culture medium, then capture images instantly via laser scanning confocal microscopy. Mean fluorescence intensity in the mitochondrial region was quantified via ImageJ software, with six independent fields of view analyzed per group.

### 5.10 JC‐1

Mitochondrial membrane potential (ΔΨ_m_) of NPCs was assessed using a JC‐1 detection kit (Beyotime, Shanghai, China, C2006). NPCs were seeded into 24‐well plates at 5 × 10^4^ cells/well. After experimental treatment, the medium was discarded, and cells were gently rinsed once with pre‐warmed (37°C) serum‐free DMEM/F12 to eliminate residual serum interference. Each well was added with 1 mL JC‐1 staining working solution, and plates were incubated in the dark at 37°C with 5% CO_2_ for 20 min. Subsequently, the staining solution was discarded rapidly, and cells were washed twice (5 min each) with pre‐cooled JC‐1 buffer to remove unbound probes. Fresh complete medium (1 mL/well) was added, and imaging was performed immediately using a laser scanning confocal microscope. At least six independent fields of view were analyzed per group, and the average value was calculated.

### 5.11 Co‐Immunoprecipitation Coupled With LC‐MS Analysis

Co‐immunoprecipitation coupled with LC‐MS/MS was utilized to screen proteins interacting with SIRT3 within NPCs. Total protein extracts from SIRT3‐stably‐overexpressing NPCs (500 µg) were incubated with 5 µg anti‐SIRT3 antibody at 4°C overnight under gentle rotation. After adding 25 µL Thermo Fisher Protein A/G magnetic beads, samples were incubated for 1 h at room temperature to capture immunocomplexes. Beads were magnetically separated, washed four times with pre‐cooled IP wash buffer, and eluted in 100 µL elution buffer for 10 min; 10 µL neutralization buffer was added immediately to collect SIRT3‐binding protein eluates. Eluates underwent 10% SDS‐PAGE electrophoresis (80 V for 30 min, then 120 V until bromophenol blue reached the gel bottom). Coomassie‐stained bands were cut and digested with trypsin at 37°C for 16 h. Digested peptides were subjected to LC‐MS/MS using a Thermo Q Exactive HF‐X instrument equipped with a 75 µm × 25 cm Acclaim PepMap RSLC C18 column. A 60 min linear gradient (5%–35% mobile phase B) was applied, where phase A was 0.1% aqueous formic acid and phase B was 0.1% formic acid in acetonitrile. PEAKS Studio 11 software was used to process raw MS data against the UniProt human database.

### 5.12 X‐Ray and MRI Examinations

At 4 and 8 weeks post‐surgery, anteroposterior and lateral radiographs of the rat caudal vertebrae were acquired using an XPERT.8 X‐ray machine (Kubtec, Stratford, CT). The seventh/eighth caudal intervertebral disc was selected as the evaluation target, and the DHI was used to quantify the intervertebral space height. Quantifications of anterior, mid, and posterior disc heights and sagittal vertebral diameters were completed with ImageJ. DHI was calculated according to the formula, and a schematic diagram of the formula is provided in the supplementary materials (Figure S4A). Philips 3.0‐T Achieva MRI equipment (Amsterdam, The Netherlands) was used to perform T2‐weighted imaging (T2WI) of the rat caudal vertebrae. Referring to the high signal characteristics of the NP region on T2WI, Pfirrmann grading was adopted to evaluate IVDD severity. The classification criteria are detailed in the supplementary materials (Table S1). All evaluations were conducted by a panel consisting of at least three orthopedic specialists.

### 5.13 Histological Analysis

At the experimental endpoint, the seventh/eighth caudal vertebral segment was rapidly dissected. Peripheral muscle and fascia were carefully trimmed to preserve the integrity of the intervertebral disc and adjacent vertebrae. Tissues were immediately fixed in 4% paraformaldehyde (PFA, Sigma‐Aldrich, St. Louis, MO) at 4°C for 48 h. After fixation, samples were transferred to 0.5 M EDTA decalcification solution for room‑temperature decalcification, with the decalcification solution refreshed daily. After sequential ethanol dehydration and xylene clearing, tissue specimens were embedded in paraffin blocks. We sectioned tissues into continuous 5 µm coronal slices, affixed them to polylysine‐coated slides, and performed a 2‐hour baking treatment at 60°C for stable attachment. All slices were processed with HE and Safranin O‐Fast Green histochemical staining, followed by immunohistochemistry (IHC) and immunofluorescence (IF) staining. Histological scoring standards are listed in the supplementary files (Table S2) [74].

### 5.14 Statistics

All statistical computations were completed via GraphPad Prism 9.0 and SPSS 26.0. All measured data were shown as mean ± SD, and n corresponds to independent biological replicates. We first performed Shapiro‐Wilk normality test and Levene's variance homogeneity test prior to subsequent statistical comparison. When contrasting two groups, we adopted an unpaired two‑tailed t‑test for normally distributed, homoscedastic data, whereas Welch's *t*‑test was chosen for heteroscedastic data. All t‐tests performed in this study were two‐tailed tests. For analyses involving three or more groups, one‐way analysis of variance (ANOVA) was run first, followed by Tukey's multiple comparisons test as the post‐hoc pairwise comparison method. Two‐way ANOVA was specially adopted for all 2 × 2 factorial rescue experiments to assess the main effects of two factors together with their interaction effects. A significant global effect (*p* < 0.05) triggered post‑hoc pairwise analysis.

## Author Contributions

**Yuxin Jin**: writing – original draft. **Yongcheng Chen**: conceptualization. **Yihao Xie**: conceptualization. **Dongyang Hu**: software. **Longhui Gong**: software. **Zhihua Chen**: data curation. **Linjie Chen**: data curation. **Minhao Gao**: validation. **Qizhu Chen**: formal analysis. **Morgan Jones**: formal analysis. **Hui Wang**: formal analysis. **Xiuling You**: formal analysis. **Kenny Yat Hong Kwan**: supervision. **Shoutao Weng**: supervision. **Taidong Lyu**: supervision. **Xiang Chen**: investigation. **Yuxiao Zhu**: methodology. **Yusheng Wang**: methodology. **Bin Li**: writing – review and editing. **Xiangyang Wang**: writing – review and editing. **Xiaofeng Jia**: writing – review and editing. **Kailiang Zhou**: writing – review and editing. **Ouqiang Wu**: writing – review and editing. **Aimin Wu**: writing – review and editing, resources.

## Ethics Statement

The human research protocol was reviewed and approved by the Medical Ethics Committee of the Second Affiliated Hospital of Wenzhou Medical University (approval number: 2024636). The animal experimental scheme obtained approval from the Laboratory Animal Ethics Committee of Wenzhou Institute, University of Chinese Academy of Sciences (approval number: WIUCAS25090201). Human nucleus pulposus specimens utilized in this work were harvested from surgical cases of the Second Affiliated Hospital of Wenzhou Medical University. Relevant human sample research procedures obtained ethical approval (Approval ID: 2024636) issued by the hospital's Medical Ethics Committee. For animal experiments, Sprague‐Dawley rats were adopted. The entire animal study plan passed ethical review by the Laboratory Animal Ethics Committee of Wenzhou Institute, University of Chinese Academy of Sciences (Ethics Reference Number: WIUCAS25090201).

## Conflicts of Interest

The authors declared no conflicts of interest.

## Supporting information




**Supporting File**: advs77870‐sup‐0001‐SuppMat.docx.

## Data Availability

The data that support the findings of this study are available from the corresponding author upon reasonable request.
