# Supplementary material for: Persistent Zn2+ Influx‐Mediated Deacetylation of ANXA2 Regulates the “Zn2+‐Autophagic Flux” and Alleviates Intervertebral Disc Degeneration
Source: Adv Sci (Weinh). 2026 Sep 27:e77870. Online ahead of print. doi: 10.1002/advs.77870 (PMC13616293; doi:10.1002/advs.77870)
Supplement: Supplementary file 1 [file ADVS-9999-e77870-s001.docx]

**Persistent Zn^2+^ influx-mediated deacetylation of ANXA2** **regulates the “****Zn^2+^-mitophagy switch” and alleviates intervertebral disc degeneration**

Yuxin Jin,^1^ Yongcheng Chen,^1^ Yihao Xie,^1^ Dongyang Hu,^1^ Longhui Gong,^1^ Zhihua Chen,^1^ Linjie Chen,^1^ Minhao Gao,^1^ Qizhu Chen,^2^ Morgan Jones,^3^ Hui Wang,^1^ Xiuling You,^1^ Kenny Yat Hong Kwan,^4^ Shoutao Weng,^1^ Taidong Lyu,^1^ Xiang Chen,^1^ Yuxiao Zhu,^1^ Yusheng Wang,^1^ Bin Li,^5^ Xiangyang Wang,^1^ Xiaofeng Jia,^6,^ ****, Kailiang Zhou,^1,^ *** Ouqiang Wu,^7,^ ** and Aimin Wu^1,^ *

^1^Department of Orthopaedics, Zhejiang-Hong Kong Joint Laboratory for Precision Diagnosis and Treatment of Spinal Diseases, Key Laboratory of Orthopaedics of Zhejiang Province, The Second Affiliated Hospital and Yuying Children's Hospital of Wenzhou Medical University, Wenzhou, Zhejiang 325000, China

^2^Department of Clinic of Spine Center, Xinhua Hospital, Shanghai Jiaotong University School of Medicine, Shanghai 200082, China

^3^Spine Unit, The Royal Orthopaedic Hospital, Bristol Road South, Northfield, Birmingham, B31 2AP, United Kingdom

^4^Department of Orthopaedics and Traumatology, Li Ka Shing Faculty of Medicine, The University of Hong Kong, 5/F Professorial Block, Queen Mary Hospital, 102 Pokfulam Road, Pokfulam, Hong Kong

^5^Orthopedic Institute, Department of Orthopaedic Surgery, The First Affiliated Hospital, School of Biology & Basic Medical Sciences, Suzhou Medical College, Soochow University, Suzhou, Jiangsu, 215006, P. R. China

^6^Department of Neurosurgery, Orthopaedics, Anatomy Neurobiology, University of Maryland School of Medicine, Baltimore, MD 21201, USA; Department of Biomedical Engineering, Anesthesiology and Critical Care Medicine, The Johns Hopkins University School of Medicine, Baltimore, MD 21205, USA.

^7^Department of Orthopaedics, Key Laboratory of Musculoskeletal System Degeneration and Regeneration Translational Research of Zhejiang Province, Sir Run Shaw Hospital, Zhejiang University School of Medicine, Hangzhou, 310000, China

*Correspondence: aiminwu@wmu.edu.cn

**Correspondence: ouqiangwu1998@163.com

***Correspondence: [zhoukailiang@wmu.edu.cn](mailto:zhoukailiang@wmu.edu.cn)

****Correspondence: Xjia@som.umaryland.edu

Conflict-of-interest statement: The authors have declared that no conflict of interest exists.

**Supplementary Materials**

The file includes:

Figs. S1 to S8

Tables 1 to 5

**FIGURES**

**Figure S1. Comparison of serum element contents among populations with different degeneration grades.** (A) Determination of peripheral blood Zn²⁺ levels in subjects of the Min-IVDD group and the Severe-IVDD group (n=30). Area under the disease-related ROC curve [AUC] = 0.807, p < 0.001. (B) Peripheral blood Zn²⁺ levels associated with different degeneration grades of IVDD (n=15). Spearman correlation analysis of the relationship between peripheral blood Zn²⁺ levels and IVDD degeneration grades (correlation coefficient [R^2^] = 0.377, p < 0.0001). (C) Determination of peripheral blood Fe²⁺ levels in subjects of the Min-IVDD group and the Severe-IVDD group (n=30). Area under the disease-related ROC curve [AUC] = 0.759, p < 0.001. (D) Peripheral blood Fe²⁺ levels associated with different degeneration grades of IVDD (n=15). Spearman correlation analysis of the relationship between peripheral blood Fe²⁺ levels and IVDD degeneration grades (correlation coefficient [R^2^] = 0.268, p < 0.0001). (E) Determination of peripheral blood Cu²⁺ levels in subjects of the Min-IVDD group and the Severe-IVDD group (n=30). Area under the disease-related ROC curve [AUC] = 0.592, p =0.220. (F) Peripheral blood Cu²⁺ levels associated with different degeneration grades of IVDD (n=15). Spearman correlation analysis of the relationship between peripheral blood Cu²⁺ levels and IVDD degeneration grades (correlation coefficient [R^2^] = 0.030, p < 0.01). (G) Determination of peripheral blood Mg²⁺ levels in subjects of the Min-IVDD group and the Severe-IVDD group (n=30). Area under the disease-related ROC curve [AUC] = 0.666, p < 0.05. (H) Peripheral blood Mg²⁺ levels associated with different degeneration grades of IVDD (n=15). Spearman correlation analysis of the relationship between peripheral blood Mg²⁺ levels and IVDD degeneration grades (correlation coefficient [R^2^] = 0.131, p < 0.005). (I) Determination of peripheral blood Ca²⁺ levels in subjects of the Min-IVDD group and the Severe-IVDD group (n=30). Area under the disease-related ROC curve [AUC] = 0.620, p =0.110. (J) Peripheral blood Ca²⁺ levels associated with different degeneration grades of IVDD (n=15). Spearman correlation analysis of the relationship between peripheral blood Ca²⁺ levels and IVDD degeneration grades (correlation coefficient [R^2^] = 0.010, p < 0.05).

**Figure S2.** **Differences in SLC39A14 content between the control group and the disease group.** (A) Heatmap of Zn²⁺ transporters differential analysis between control and IVDD groups revealed that SLC39A14 exhibited the highest expression level and the most significant intergroup differential expression. (B) Volcano plot for screening upregulated and downregulated differentially expressed genes (Log FC > 1, p < 0.05). (C) Gene Ontology (GO) analysis between the IVDD group and the control group revealed that the enriched terms were Zinc & Ion Homeostasis, Mitochondrial Stress & Hypoxia, ECM & Cartilage Degeneration, and Inflammation & Signaling. (D) Immunofluorescence staining of SLC39A14 in human NP tissues from the control group and the degenerative group (n=6, Scale bar: 50 μm). (E) Comparison of SLC39A14 mRNA expression differences between the control group and the IVDD group (n=6). (F) Analysis of SLC39A14 protein expression between the control group and the degeneration group (n = 3). (G) Differences in intracellular SLC39A14 mRNA expression in nucleus pulposus cells after the addition of the in vitro stimulus mimic TBHP (n=6). (H) Differences in intracellular SLC39A14 protein expression in nucleus pulposus cells after the addition of TBHP (n=3). (I, J) The silencing efficiency of SLC39A14 was verified by WB and qPCR. (K) FluoZin™-3 detection of intracellular Zn²⁺ changes between the control group and the TBHP group (n=6, Scale bar: 20 μm). (L) FluoZin™-3 detection of intracellular Zn²⁺ changes between the LV-shNC group and the LV-shSLC39A14 group (n=6, Scale bar: 20 μm).

**Figure S3.** **Functional verification of the SLC39A14 channel.** (**A**) Marker genes and biological functions of NPC subpopulations. (**B**) Detection of intracellular Zn²⁺ levels in different treatment groups using FluoZin™-3 (green: Zn²⁺; red: actin). (**C**) Comparison of intracellular autophagic changes among different groups after transfection with mCherry-LC3/GFP-LC3 dual-labeled lentivirus. (**D**) Supplementary quantitative analysis of P62 (** = 0.0011, ** = 0.0040, ns = 0.9974; n = 3) and ATG5 (** = 0.0095, **** < 0.0001, *** = 0.0004, ns = 0.7385; n = 3) under different treatments. (**E**) Quantitative analysis of LC3 expression and mitochondrial length (n = 6). (**F**) SA-β-gal staining to assess the ratio of senescence-positive cells (n = 6; Scale bar: 100 μm). (**G**) Differences in protein levels of senescence-related proteins (TP53, CDKN1A, CDKN2A) among cells after different treatments (n = 3). (**H**) Differences in protein levels of SASP-related proteins (TNF-α, IL-1β, IL-6) among cells after different treatments (n = 3). (**I**) Mitochondrial Zn²⁺ was detected via dual fluorescent staining with RhodZin-3 AM and MitoTracker (n = 6; Scale bar: 10 μm). (**J**) Fluorescence results of mCherry-GFP-LC3 in each group treated with CQ (n = 6; Scale bar: 20 μm). Note: mitophagy flux (mCherry-LC3/GFP-LC3).

**Figure S4.** **In vivo safety evaluation of Zn²⁺ and in vitro functional verification of its downstream factors.** (A) Histological Section Images of Heart, Liver, Spleen, Lungs and Kidneys in Rats after Regular Zinc Diet and High-Zinc Diet Intervention (n = 6; Scale bar: 200 μm). (B) HE staining, SO staining, IHC staining for SIRT3, and tissue immunofluorescence staining of caudal vertebral tissue sections from rats fed a regular zinc diet and a high-zinc diet (n = 6; Scale bar: 20 μm for HE, SO, and IHC staining; Scale bar: 20 μm for tissue immunofluorescence staining). (C) Total acetylation levels in the control group and degenerated group after zinc gluconate (ZnG) treatment (n = 3). (D) Changes in the expression of SIRT family proteins following ZnG treatment (n = 3). (E) Evaluation of mitochondrial morphology and mitochondrial membrane potential (Δψm) after TBHP treatment and SIRT3 overexpression via MitoTracker and JC-1 staining (n = 6; MitoTracker: Scale bar: 20 μm; JC-1: Scale bar: 50 μm). (F) WB analysis to detect the activation levels of mitophagy-related proteins PINK1 and Parkin following SIRT3 overexpression (n = 3). (G) Measurement of Intervertebral Space Height Using Disc Height Index (DHI) on X-ray Images. Note: static marker levels (Mitotracker Red, JC-1, PINK1, Parkin).

**Figure S5. Supplementary verification of the binding of ANXA2 to mTOR and the function of ANXA2.** (A) SASA plot indicates strengthened ligand-receptor interaction after acetylation. (B) DSSP-based secondary structure analysis of ANXA2 WT and ANXA2_Ac in complex with mTOR. (C) RMSF plots of ANXA2 in complexes with ANXA2 WT and ANXA2_Ac. (D) Immunofluorescence staining revealed the colocalization of mTOR and ANXA2 (n = 6; Scale bar: 50 μm). (E) Acetylation levels of ANXA2 in different degeneration grades (n = 3). (F) The binding rate between ANXA2 and mTOR gradually increases with the extension of TPEN addition time. (G) Subcellular fractionation of cytoplasmic and mitochondrial proteins was performed to detect the differential expression of ANXA2 between the control and IVDD groups (n = 3). (H) Fluorescence co‑localization of SIRT3 and ANXA2 (n = 6; Scale bar: 50 μm). (I) Effect of SLC39A14 on downstream proteins regulated by ANXA2 (n = 3). (J) Effect of SIRT3 overexpression on p-4EBP1/4EBP1 and p-S6K/S6K in ANXA2 WT and K302R mutants (n = 3).

**Figure S6.** **Functional role of the ANXA2 K302 Site in mitophagy and cellular senescence.** (A) Analysis of the cytoplasmic and nuclear localization levels of TFEB in WT and K302R cells before and after SIRT3 overexpression (n=3). (B) Detection of mitochondrial reactive oxygen species (ROS) levels in WT and K302R cells before and after SIRT3 overexpression using MitoSOX (n=6, Scale bar: 100 μm). (C) Detection of mitochondrial morphology and autophagic protein (LC3) levels in WT and K302R cells before and after SIRT3 overexpression via MitoTracker and LC3 double fluorescence staining (n=6, Scale bar: 20 μm). (D) JC-1 staining to detect the regulatory effect of SIRT3 on mitochondrial membrane potential (Δψm) in WT and K302R cells (n = 6; Scale bar: 50 μm). (E) Transmission electron microscopy (TEM) was used to detect mitochondrial morphology in different treatment groups after TBHP stimulation (n = 6; Scale bar: 500 nm, arrows indicate mitochondria, and asterisks mark autophagosomes). (F) Overexpression of SIRT3 in WT and K302R mutant cells, followed by detection of the expression of senescence-related proteins (TP53, CDKN1A, CDKN2A) and SASP-related secretory proteins (TNF-α, IL-1β, IL-6) (n = 3). (G) SA-β-gal staining to detect the positive ratio of senescent cells in ANXA2 WT and K302R cells after different treatments (n = 6; Scale bar: 100 μm). Note: Static marker levels (TEM, Mitotracker Red, JC-1, MitoSOX).

**Figure S7.** **Staining analysis of animal tissue sections after Zn²⁺ content interference.** (A) IHC staining for detecting the expression levels of AXAN2, and mTOR in tissue (n = 6; Scale bar = 1 mm). (B) Heat maps were plotted based on NP shape, NP area, cell number, cell morphology, border appearance, lamellar organization, tears/fissures/disruptions, disruptions/microfractures as well as osteophyte/ossification. (C) Quantitatively analyze the proportion of positive cells in immunohistochemistry (n = 6).

**Figure S8.** **ANXA2 is metabolized in cells via the autophagolysosomal pathway.** (A) Time-dependent changes in ANXA2 protein levels after combined lysosomal inhibition with Leupeptin (Leu, a serine and cysteine protease inhibitor) and NH_4_Cl (a lysosomal acidification inhibitor) (n = 3). (B) Detection of ANXA2 protein expression levels after SIRT3 overexpression combined with the degradation inhibitor N/L (n = 3). (C) ANXA2 protein levels after rapamycin (a specific inhibitor of mammalian target of rapamycin (mTOR)) treatment (n = 3). (D) Changes in ANXA2 content after inhibition of lysosomal function by CQ (a classic lysosomal inhibitor). (E) Comparison of differences in ANXA2 content relative to the control group after inhibiting lysosomal pathway degradation with CQ and de novo protein synthesis with CHX (n = 3).

**TABLES**

**Table S1.** Pfirrmann classification

**Table S2.** Histological grading scale

**Table S3.** Primary antibodies used in this study

**Table S4.** Primers used for qPCR

**Table S5.** Construction strategies of shRNA and siRNA
